# The Correlation between Chemical Structures and Antioxidant, Prooxidant, and Antitrypanosomatid Properties of Flavonoids

**DOI:** 10.1155/2017/3789856

**Published:** 2017-07-02

**Authors:** João Luiz Baldim, Bianca Gonçalves Vasconcelos de Alcântara, Olívia da Silva Domingos, Marisi Gomes Soares, Ivo Santana Caldas, Rômulo Dias Novaes, Tiago Branquinho Oliveira, João Henrique Ghilardi Lago, Daniela Aparecida Chagas-Paula

**Affiliations:** ^1^Chemistry Institute, Federal University of Alfenas (Unifal-MG), 37130-001 Alfenas, MG, Brazil; ^2^Human and Natural Sciences Centre, Federal University of ABC (UFABC), 09210-580 Santo André, SP, Brazil; ^3^Parasitology and Pathology Department, Federal University of Alfenas (Unifal-MG), 37130-000 Alfenas, MG, Brazil; ^4^Department of Structural Biology, Federal University of Alfenas (Unifal-MG), 37130-000 Alfenas, MG, Brazil; ^5^Department of Pharmacy, Federal University of Sergipe (UFS-SE), 491000-000 São Cristóvão, SE, Brazil

## Abstract

Flavonoids have demonstrated in vivo and in vitro leishmanicidal, trypanocidal, antioxidant, and prooxidant properties. The chemotherapy of trypanosomiasis and leishmaniasis lacks efficacy, presents high toxicity, and is related to the development of drug resistance. Thus, a series of 40 flavonoids were investigated with the purpose of correlating these properties via structure and activity analyses based on integrated networks and QSAR models. The classical groups for the antioxidant activity of flavonoids were combined in order to explain the influence of antioxidant and prooxidant activities on the antiparasitic properties. These analyses become useful for the development of efficient treatments for leishmaniasis and trypanosomiasis. Finally, the dual activity of flavonoids presenting both anti- and prooxidant activities revealed that the existence of a balance between these two features could be important to the development of adequate therapeutic strategies.

## 1. Introduction

Protozoan parasites of the Trypanosomatidae family are the etiological agent of several significant neglected tropical diseases. The flagellated protozoan parasite *Trypanosoma cruzi* causes Chagas disease, also known as American trypanosomiasis. It is estimated that over six million people are infected with *T. cruzi* worldwide, and this disease represents one of the important health problems in South, Central, and North America [1, 2]. Chagas disease causes almost 12000 deaths annually, while the number of diagnosed cases has increased in nonendemic regions, due to emigration events [3, 4].

The Human African trypanosomiasis or sleeping sickness, a fatal and neglected tropical disease, is caused by two parasites *Trypanosoma brucei gambiense* and *Trypanosoma brucei rhodesiense*. This disease is transmitted by tsetse fly (genus *Glossina*), a vector which is only found in tropical Africa, this explains the *T. brucei* geographic restriction [5, 6].

In the other concern, leishmaniasis is a complex of diseases caused by different species of the protozoan parasite *Leishmania* sp. These parasites infect macrophages and cause a wide spectrum of symptoms ranging from cutaneous lesions to potentially fatal visceral infections. *Leishmania donovani* is the causative agent of visceral leishmaniasis, which is fatal in the absence of treatment [7].

There is no effective vaccine for all these diseases, and its regulation is based on vector control and chemotherapy [8]. The drugs currently used have high toxicity and limited efficacy and demand a long period of treatment [4, 8]. Besides, there are evidences of development of drug resistance by the parasites [8–10]. Thus, it is important to investigate new drugs or compounds that could be associated with the traditional treatment.

Flavonoids have both antioxidant and prooxidant properties potentially relevant to the treatment of those parasitic diseases [8, 11]. They are phenolic substances, which constitute a major class of secondary metabolites, formed from the aromatic amino acids phenylalanine and tyrosine, and malonate [12]. Flavonoids are usually found in plant food and fruits [13, 14] and have demonstrated numerous biological properties, including vasoprotective, anti-inflammatory, antihepatotoxic, and anticarcinogenic actions, effects frequently linked to their antioxidant properties [15]. Antioxidant activity can occur by different mechanisms, such as sequestration of oxidant agents, scavenging of free radicals, altering the expression of the multiple genes encoding the enzymes with antioxidant function, and change of cell signalling. According to the surrounding medium acidity, flavonoids can be partially or totally ionized, allowing the participation of ions in the antioxidant actions [16].

Additionally, flavonoids are also considered safe compounds with low potential to induce organic toxicity [17]. Conversely, they can exhibit prooxidant activity, explaining the mutagenic [14], cytotoxic [18], and toxic effect against parasites [19, 20]. Prooxidant and antioxidant properties of flavonoids depend on the environment in which they are inserted and their chemical structure and concentration [11, 13].

Several flavonoids were systematically investigated against *L. donovani*, *T. cruzi*, and *T. brucei*, showing interesting results from in vitro and in vivo experiments [21]. However, it remains unclear to what extent the molecular structure and oxidant potential of flavonoids are associated with their antiparasitic effects. Thus, the aim of the present study was to investigate whether there is an association between a series of 40 flavonoids and their antiparasitic, antioxidant, and prooxidant properties. The use of multivariate statistical analysis related to this investigation can help to understand the function of the dual activity of flavonoids as anti- and prooxidant compounds and how they are involved in the mechanisms by which these molecules act against trypanosomatids. Besides, this can be useful for the development of efficient treatments for leishmaniasis, sleeping sickness, and Chagas disease.

## 2. Materials and Methods

### 2.1. Flavonoid Dataset

The flavonoid dataset was created according to the results from Tasdemir et al. [21] for the in vitro antitrypanosomal and antileishmanial activities. The antioxidant potential of all flavonoids investigated was determined from their Trolox equivalent antioxidant capacity (TEAC). TEAC values for each flavonoid were based on previous studies in vitro (Table 1 for references). The antiparasitic activities were determined from the half maximal inhibitory concentration (IC_50_) reported in previous in vitro assays [21]. IC_50_ and TEAC, originally obtained in *μ*g/mL and mM, respectively, were transformed to molar (M) and normalized as pIC_50_ values (−logIC_50_) in order to facilitate comparisons. Higher values of pIC_50_ indicate antiparasitic/antioxidant activities (Table 1).

### 2.2. Network

**Figure 1:**
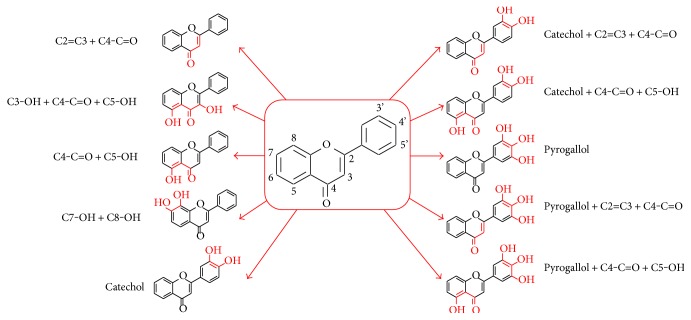
The main subunit combinations highlighted in red. The combinations were correlated to the biological activities for the selection of the best chemical features which make flavonoids active against trypanosomatid species investigated in this work.

The network was created by using the stand-alone software Cytoscape 3.3.0 [22]. The network file was created according to the software requirements and using a dataset containing flavonoid names; pIC_50_ against *L. donovani*, L6 cells, *T. cruzi*, *T. brucei*, and TEAC; and structure combinations (main features denominated as combinations can be checked in Figure 1). Different attributes, based on the pIC_50_ values, were used to create each network subset. The clustering algorithm, AllegroLayout 2.2, using the Spring-Electric layout algorithm, was adopted for all analyses. The subnetworks were created according to the visual parameters correlated to the pIC_50_ values.

### 2.3. Venn Diagram

The Venn diagram was created by using the online tool Venny 2.1 (http://bioinfogp.cnb.csic.es/tools/venny/) for four groups of combinations elected according to the number of shared characteristics. Venny diagram output: style colours, show percentage. Shared elements were explained using Cytoscape subnetworks.

### 2.4. Heatmaps

The heatmaps were built using the software Gitools 2.3.1 [23], based on their biological activities against *L. donovani*, *T. brucei*, *T. cruzi*, and L6 cell toxicity. The data treatment was based on several matrices containing different sets of information, depending on the interpretation. Hierarchical clustering analysis (HCA) was carried out for columns using Euclidean distance. Color scales were adopted for each individual case based on yellow (lower values of pIC_50_), red (intermediate values of pIC_50_), and black (higher values of pIC_50_). The correlation values of all flavonoids with the most active compounds were calculated based on their pIC_50_.

### 2.5. Molecular Descriptors

The values for the molecular descriptors to the flavonoid series were determined using the software PaDEL version 2.2.1 [24]. The three-dimensional geometries were calculated and optimized by the PM7 semiempirical method with the software MOPAC2016 [25, 26]. The descriptor data were normalized with the software Weka 3.8.0 using the scale 1 and translation 0 [27, 28]. After the normalization, the important descriptors for each property (based on the values of IC_50_) were selected using the CfsSubsetEval as an attribute evaluator (standard parameters: P-1, E-1) and the search method Best First (standard parameters: D-1, N-5). The CfsSubsetEval evaluates the worth of a subset of attributes by considering the individual predictive ability of each feature along with the degree of redundancy between them [28]. The Best First algorithm evaluates the importance of attributes by correlation-based heuristic function. It searches the space of attribute subsets by greedy hill climbing augmented with a backtracking facility. Those subsets of descriptors, which show high correlation with the property (class) and also have lower intercorrelation, are preferred. The search space was explored with the Best First search strategy using forward selection with a stopping criterion of five consecutive fully expanded nonimproving subsets. Initial search starts with an empty set of features that has a merit = 0. The subset with the highest merit is reported; higher values are better [28–30]. A merit is given by the following equation: ${Merit}_{s}=N\overset{¯}{pij}/\surd \left[N+N\left(N-1\right)\;\overset{¯}{pjj}\;\right]$, where Merit_*s*_ is the heuristic merit of a feature subset _*s*_ containing *N* features, pij is the mean feature class correlation, and pjj is the average feature-feature intercorrelation [30]. The following merits of the best subset from attribute selection were found: 0.89, 0.91, 0.86, 0.92, and 0.72, respectively, correlating to the following activities against *L. donovani*, *T. brucei*, *T. cruzi*, TEAC, and L6. Additionally, descriptors that describe the same molecular characteristics through different calculations were excluded, keeping only one descriptor by a physicochemical feature per activity (Table S4 available online at https://doi.org/10.1155/2017/3789856), as in the case of TDB4e (3D topological distance-based autocorrelation—lag 4/weighted by Sanderson electronegativities) and minHBd (atom-type electrotopological state calculated by the minimum E-states for strong hydrogen bond donors) which both describe an electrotopological feature. These strategies for selection of descriptors are crucial to get the quality of the model development and commonly used in QSAR studies [29, 31–33]. In this sense, up to six descriptors were used to build the prediction models of activity against *L. donovani* (Ki, AATS7i, GATS8v BCUTp-1h minHCsatu, and TDB1r), *T. brucei* (ATSC6c, MATS8s, VR1_Dzp, TDB6i, RDF55p, and E2s), *T. cruzi* (C1SP2, SHBint5, TDB4m, TDB9v, TDB4e, and Dp), TEAC (ATSC3i, MATS1c, GATS3p, VR2_Dzs, BCUTc-1h, and AVP-1), and L6 cells (VR2_Dzv, MDEC-11, minHBint5, maxHBint5, and CIC5). Detailed information about the data from these descriptors can be found at the supplementary session (Table S4 and Table S5–S9, resp.).

### 2.6. Prediction Models

The descriptors selected above were used for building artificial neural network (ANN) for certification of the quantitative structure-activity relationship (QSAR). The ANN were built on Weka 3.8.0 [27, 28], based on normalized descriptor data and values of IC_50_. The data were normalized using the software Weka 3.8.0 scaling 1–0. The classifier multilayer perceptron was used, and the parameters were optimized for each activity: *L. donovani* (L, 0.6; M, 0.1; N, 500; S, 0; E, 20; and H, 1), *T. brucei* (L, 0.3; M, 0.2; N, 800; S, 0; E, 20; and H, 1), *T. cruzi* (L, 0.5; M, 0.2; N, 600; S, 0; E, 20; and H, 6), TEAC (L, 0.1; M, 0.1; N, 400; S, 0; E, 20; H, 3), and L6 (L, 0.3; M, 0.2; N, 300; S, 0; E, 20; and H, 3) (Figure S1). The meaning of each parameter is as follows: L—learning rate, the amount of the weights is updated; M—momentum applied to the weights during updating; N—training time, the number of epochs to train through; S—seed used to initialize the random number generator; E—validation threshold used to terminate validation; and H—the hidden layers of the network. The multilayer perceptron uses backpropagation for determining the weights [28, 29]. In each node (or neuron), the sigmoid function is used, and the weight for each node is calculated for a perceptron with one hidden layer by derivation. The weight is calculated for every training instance. The changes are associated with values regulated during the tuning of the neural network as momentum, learning rate, training time, and number of hidden layer. The weight of the previous instance is multiplied by the learning rate, and the outcome is then subtracted from the next value of weights. Because of this mechanism, this version of generic gradient descent strategy is called backpropagation [28, 29]. The values of *R*^2^ (squared correlation coefficient) were evaluated to measure the goodness of fit of the models (using all 40 flavonoids in Table 1); the values of *Q*^2^ (squared correlation coefficient for cross-validation) were determined on 10-fold cross-validation to evaluate the robustness, validity of the models, and internal predictivity (using all 40 flavonoids in Table 1); and the values of *P*^2^ (squared correlation coefficient for test set) were determined on split 65% of the dataset to train the model (26 flavonoids randomly chosen by the software Weka 3.8.0) and test the remainder (14 flavonoids, Table S10) to do external validation with data not used in the ANN model development. Besides, the scramble test was performed with the bioactivities randomly changed on the dataset to do an additional validation for the models.

## 3. Results and Discussion

Since trypanosomatids (*Trypanosoma* and *Leishmania*) present a limited antioxidant system, essentially dependent on trypanothione reductase, these parasites experience difficulties in neutralizing reactive oxygen (ROS) and nitrogen (RNS) species [34, 35]. Thus, prooxidant drugs (i.e., benznidazole (BNZ) and nifurtimox (NFX) against *T. cruzi*; Pentostam® and Glucantime® against *Leishmania* sp.) are commonly used in the treatment of trypanosomiasis and leishmaniasis, especially due to their ability to induce parasite lipid, protein, and DNA oxidation from production of highly toxic reactive molecules (i.e., nitric oxide (NO), anion superoxide (O_2_^·−^), hydrogen peroxide (H_2_O_2_), and hydroxyl radicals (OH^−^)), determining parasite death [36]. BNZ and NFX act to cause the production of highly toxic oxygen species, such as superoxide, hydrogen peroxide, and hydroxyl radicals. In the case of leishmaniasis, the compounds Pentostam and Glucantime were also considered prooxidant agents, because the proposed mode of action is the inhibition of trypanothione reductase and induction of the efflux of thiols [37].

From the understanding that the antioxidant enzymatic system is less evolved in trypanosomatids than mammalian hosts [9], prooxidant compounds, including flavonoids, can be rationally selected and used as candidate molecules in the treatment of leishmaniasis, sleeping sickness, and Chagas disease.

The chemical structure of each flavonoid is related to their antioxidant and prooxidant properties [11, 38], as well as antiparasitic properties [21]. The previous published data about these properties of 40 flavonoids representative of the main classes are allocated in Table 1.

In the present study, combined chemical features (Figure 1) with the most important antioxidant potential were used to classify the activities of individual flavonoids. This investigation leads into a consideration of the Bors' criteria [39] in addition to peculiarities of the flavonoid series. The chemical features were combined as follows: catechol (ring B: 3′,4′-diOH), pyrogallol (ring B: 3′,4′,5′-triOH), C2=C3 + C4-C=O (double bond between C2-C3 and the presence of carbonyl on the carbon 4), C4-C=O + C5-OH (carbonyl on the carbon 4 and the presence of –OH on the carbon 5), C7-OH + C8-OH (presence of –OH group on the carbons 7 and 8), C3-OH + C4=O + C5-OH (presence of a carbonyl group and –OH groups on the carbon 3 and 5), catechol + C2=C3 + C4-C=O, catechol + C4-C=O + C5-OH, pyrogallol + C2=C3 + C4-C=O, and pyrogallol + C4-C=O + C5-OH. Single substituents were excluded from the group combination analyses using only the well-established groups considered important to the antioxidant activity (Figure 1) [40].

**Figure 2:**
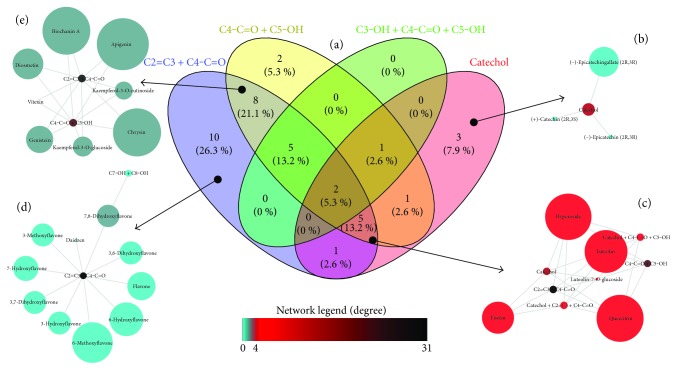
The overview in combination distribution and biological activities for the flavonoid series. (a) The Venn diagram exhibiting the distribution of moieties combinations for 38 flavonoids investigated. The groups of substitutions were adopted following Bors' criteria with modifications according to the flavonoid peculiarities. (b) The elements included exclusively in the conjunction catechol (nodes colored and sized according to *L. donovani* pIC_50_). (c) The five common elements presenting C4-C=O + C5-OH, C3-OH-C4-C=O + C5-OH, and catechol as shared characteristics (nodes colored and sized according to *L. donovani* pIC_50_). (d) The ten flavonoids included exclusively in the conjunction C2=C3 + C4-C=O (nodes colored and sized according to TEAC pIC_50_). (e) The eight flavonoids sharing the C2=C3 + C4-C=O and C4-C=O + C5-OH groups (nodes colored and sized according to *T. brucei* pIC_50_). The open-source online software Venny 2.1.0 was used for building the Venn diagram. The subsets of the original network were obtained from Cytoscape 3.3.0. The legend indicates the degree of correlation for both compounds to their respective groups and groups to their respective compounds. Conjunction colors follow the legend colors. Arrows indicate unique or shared subunit combinations.

A Venn diagram (Figure 2(a)) was generated to explain the distribution of the combinations for the flavonoids, attempting to elucidate the influence of certain chemical groups on the biological activities. In addition, a network profile was created to correlate the most important subunit combinations, according to the most important groups, to the antioxidant activity associating them to the antiparasitic activities. The node sizes represent the pIC_50_ values of each biological activity investigated (Figure 2).

Three flavonoids contain exclusively the catechol group: (−)-epicatechin, (−)-epicatechin gallate, (+)-catechin, corresponding to 7.9% of the flavonoids. Flavonoids with only the catechol group did not have correlation to higher pIC_50_ values against the parasitic diseases investigated suggesting that its existence is essential only to the antioxidant activity (Figure 2(b)) [40]. These three compounds lack the double bond between C2 and C3. The existence of the Δ^2, 3^ double bond was widely spread on the 40 flavonoids (31 of 40); however, its presence was common to all active flavonoids. A very important characteristic to the prooxidant activity of flavonoids is the existence of the Δ^2, 3^ double bond [11], suggesting that this double bond could be involved in the mechanism of action of these compounds on trypanosomatids.

The moiety pyrogallol itself was not effective for the biological activities against trypanosomatids. (−)-Epicatechin gallate and (−)-epigallocatechin gallate share the 3′,4′,5′-trihydroxy moiety and did not participate in the Venn diagram analysis due to their exclusive characteristic. These compounds did not exhibit significant pIC_50_ values, being more active against *T. brucei* with cytotoxicity levels close to the biological activities: (−)-epicatechin gallate (pIC_50_: 4.3), (−)-epigallocatechin gallate (pIC_50_: 4.7), and L6 cells (pIC_50_: 3.7 and 4.5 resp.).

The flavonoid eriodictyol, which has the C4-C=O + C5-OH combined to the catechol group, exhibits higher pIC_50_ against the parasites investigated (especially *T. cruzi*), compared to those compounds only with the catechol group. On the other hand, the flavonoid luteolin, most structurally similar to eriodictyol, has its biological activity against *T. brucei* and *L. donovani* positively affected by the presence of the Δ^2,3^. Conversely, eriodictyol was less toxic and more active against *T. cruzi*, presenting a higher antioxidant activity compared to that of luteolin.

Five compounds shared the combinations C2=C3 + C4-C=O + C5-OH + catechol: hyperoside, luteolin, luteolin-7-*O*-glucoside, quercitrin, and rutin, corresponding to 13.2% of the flavonoids investigated, and have carbohydrate subunits on C3, C5, or C6. Luteolin and its 7-*O*-glucoside derivative have the higher pIC_50_ against *L. donovani*. However, luteolin presents the higher L6 cell toxicity between these five flavonoids (Figure 2(c)). The luteolin-7-*O*-glucoside showed to be the less active against *T. cruzi* and *T. brucei*, suggesting that the glucoside moiety in position 7 decreases the biological activity against *Trypanosome* strains in comparison to 3-*O*-galactose (hyperoside), 5-*O*-rhamnose (quercitrin), and 3-*O*-rutinose (rutin). The effective groups of the antioxidant activities alone were not directly correlated to the antiparasitic activities. However, the examination of their subunit combinations leads to the identification of common characteristics of active compounds as in the case of rutin, rhamnetin, quercitrin, and hyperoside flavonoids that share the same subunit combinations C2=C3 + C-C4=O, C4-C=O + C5-OH, and catechol with only one difference to rhamnetin, which has the hydroxyl group on C3. This interpretation only took into consideration the flavonoid nucleus, since the carbohydrate position was discussed above. These compounds also have similar activity against *T. cruzi* and the most active was rhamnetin.

The compound fisetin, the only one with the combination C2=C3 + C4-C=O + catechol (2.6% of all flavonoids investigated), shared characteristics with the most active group of compounds against *L. donovani* (fisetin, luteolin, luteolin-7-*O*-glucoside, and quercetin). Nevertheless, the three last compounds also share the combination C2=C3 + C4-C=O + C5-OH + catechol which also contributes to their higher pIC_50_ values against *L. donovani*, *T. brucei*, and *T. cruzi*. The presence of the C2=C3 + C4-C=O combination corresponded to 26.3% of all flavonoids, and this conjunction did not explain the antiparasitic activity itself probably due to their different hydroxyl substitution patterns. Considering the TEAC results, the flavonoids 6-methoxyflavone, 3-methoxyflavone, and 7-hydroxyflavone presented the higher pIC_50_. However, the other flavonoids containing only the C2=C3-C4-C=O group did not reach higher antioxidant activity (Figure 2(d)). This group is correlated to some of the best pIC_50_ values against *T. cruzi*. The addition of a single hydroxyl group on C5 (resulting in a double bond between C2 and C3, carbonyl on C4, and hydroxyl group on C5) presented intermediate values against *T. cruzi*, *T. brucei*, *L. donovani* (eight compounds presented this characteristic, corresponding to 21.1% of all combinations: apigenin, biochanin A, chrysin, diosmetin, genistein, kaempferol-3-*O*-glucoside, kaempferol-3-*O*-rutinoside, and vitexin) (Figure 2(e)). On the other hand, the addition of another hydroxyl on C3 displaying a C2=C3-OH + C4-C=O + C5-OH pattern (five compounds shared this characteristic, corresponding to 13.2% of all combinations: galangin, isorhamnetin, kaempferol, morin, and myricetin) resulted in an intermediate-to-high pIC_50_ values against *T. cruzi*, *T. brucei*, and *L. donovani*. Likewise, their pIC_50_ for L6 cells were similar, excluding morin, which presented the lower toxicity of this series.

Two compounds sharing all characteristics, corresponding to 5.3% of all combinations: quercetin and rhamnetin, presented intermediate-to-high pIC_50_ values against trypanosome strains with rhamnetin owing the best activity. In addition, the toxicity of L6 cells was lower in the flavonoid rhamnetin. The biological activity of these compounds against *L. donovani* presented an opposite behaviour compared to the effect on trypanosome strains, in which quercetin was the most active flavonoid. The presence of only C4-C=O + C5-OH (two compounds, corresponding to 5.3% of all combinations: hesperidine and naringenin) resulted in intermediate-to-low biological activity against all parasites (Figure 2(a)).

**Figure 3:**
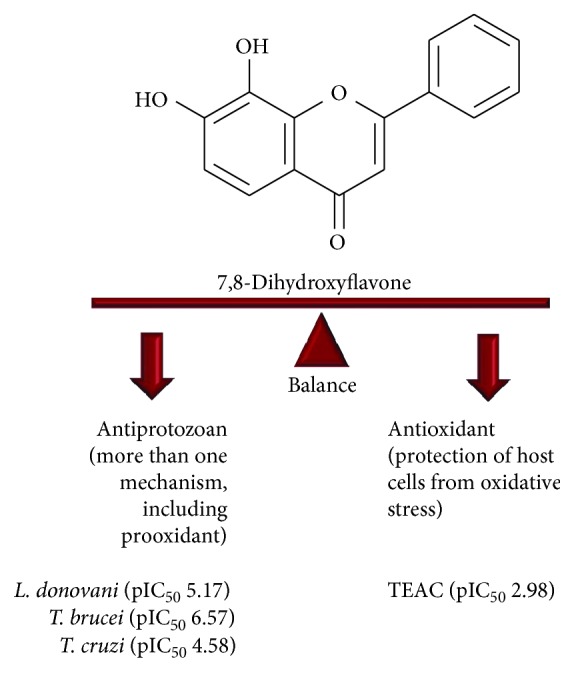
The balance between prooxidant and antioxidant properties and its dual importance to the antiparasitic activity of flavonoids in the treatment of trypanosomatid infections.

Thus, the antioxidant activity of the 40 flavonoids studied in this work presented no direct correlation to any parasite or L6 cell cytotoxicity in the means of TEAC assay. The Pearson correlation scores obtained from pIC_50_ values were considered weak-to-moderate correlations (TEAC versus *L. donovani* (*r* = −0.27), TEAC versus *T. brucei* (*r* = −0.21), TEAC versus *T. cruzi* (*r* = 0.19), and TEAC versus L6 cells (*r* = −0.34)). These results are in accordance to the expected behaviour of antiparasitic drugs, since induction of oxidative stress is a key point to eradicate trypanosomatids [35]. Meanwhile, the antioxidant property of flavonoids could protect the host facing an infection, since it could attenuate the reactive tissue damage in parasitized organs and the excessive production of proinflammatory mediators such as nitric oxide and prostaglandins [41]. In this sense, it is more expected that effective antiparasitic compounds present a balance between prooxidant and antioxidant activities, especially due to the rudimentary antioxidant system observed in trypanosomatids compared to their mammalian hosts (Figure 3) [4]. All flavonoids investigated in this work are antioxidants (Table 1), and those which also present prooxidant activity (data on the supplementary session Table S3) could be useful for treatment against parasites.

It is important to highlight that trypanothione reductase (TryR) and superoxide dismutase (SOD) integrate the antioxidant system of trypanosomatids. However, enzymes involved in the redox metabolism such as catalase (CAT) and glutathione peroxidase (GPX) are absent in these group of parasites. These enzymes are important to protect the parasite from oxidative stress, since the TryR seems to be the only mechanism to detoxify hydrogen peroxide species [9]. Besides, TryR is essential for the trypanosomatids and is much less efficient than the mammalian glutathione peroxidase. Thus, these protozoan parasites are highly more susceptible to stress oxidation than the mammalian species [9].

Conversely, *T. cruzi* strains possess two peroxiredoxins, an ascorbate-dependent hemoperoxidase and several distinct peroxidases. Although the activity and levels of antioxidant enzymes (i.e., ascorbate peroxidases and TryR) are more pronounced in the virulent strains or multiresistant strains, these aspects remain stable during different parasite life stages, characteristics potentially related to infectivity and pathogenicity observed in different parasite strains. Considering other types of resistance, stress-induced oxidant resistance in *L. chagasi* is suggested to be associated with heat shock proteins, which do not increase ROS scavengers [10]. Additionally, the exact mechanism by which *T. cruzi* resists to the oxidative burst triggered by mammalian macrophages is still unknown. However, it was suggested that the resistance could occur by inhibition of macrophage activity and escape of the endosomal-lysosomal system [10]. Thus, beyond the chemical characteristics of the molecules used, the susceptibility of trypanosomatids to prooxidant drugs seems to be directly related to the species and parasite strains.

In this context, antioxidant property of flavonoids might protect the host from the side effects of the common prooxidant drugs (i.e., BZN and NFX) [1, 42]. Furthermore, it could attenuate the secondary oxidative stress triggered by the defence cells against the infectious agent, which is also very toxic for neighbouring host tissues [20, 34, 37]. At the same time, the prooxidant activity is one of the mechanisms of action of flavonoids against protozoan parasites [19, 35]. Flavonoids can increase oxidative stress in the parasite, accepting electrons from oxidoreductases that are unique to the parasite, acting as prooxidant in this case [35].

Some features for the effective prooxidant activity of flavonoids were well established. There is evidence that this activity is directly proportional to the total number of hydroxyl groups in a flavonoid structure and its concentration. The prooxidant activity could be important in vivo whether free transition metal ions participate in oxidation processes and might be important to certain metal overload diseases [11]. The reaction of O_2_ with transition metal ions and free radicals results in a variety of reactive oxygen and nitrogen species (ROS and RNS), which can effectively initiate reactions and attack biomolecules [11]. The double bond between C2 and C3 is very important to this activity, as well as, at least one hydroxyl substituent. Other important feature established is that the *O-*methylation of the hydroxyl groups can inactivate both prooxidant and antioxidant properties of the flavonoids.

**Figure 4:**
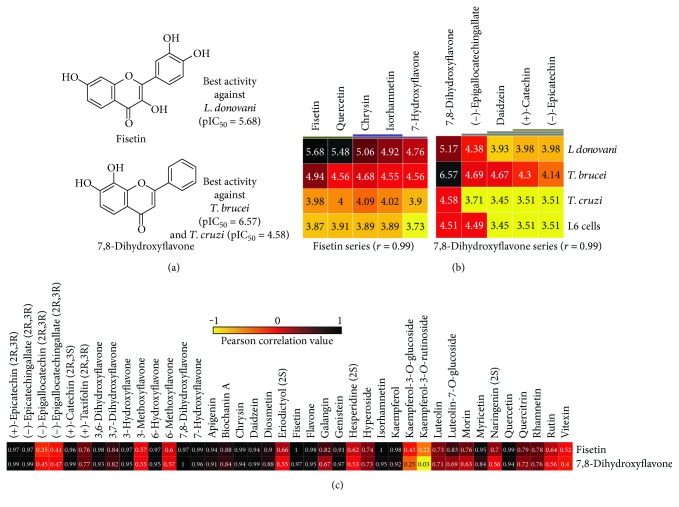
Results of hierarchical clustering analyses carried out in Gitools 2.3.1 using Euclidean distance and correlation analysis with the most active flavonoids. (a) The most active compounds: fisetin against *L. donovani* and 7,8-dihydroxyflavone against *T. brucei* and *T. cruzi*. (b) The most relevant part of HCA cluster based on pIC_50_ of all flavonoids. (c) Correlation values of the 40 flavonoids studied with the more active compounds. The intensity of the colors in the heatmap is according to the pIC_50_ values.

The pIC_50_ of each flavonoid in this study for each parasite were investigated with the purpose of finding a common behaviour between the best compounds against *L. donovani*, *T. brucei*, and *T. cruzi* and their biological activity (Figure 4). The most active compound against *L. donovani*, fisetin (pIC_50_ = 5.68), and the most active compound against *T. brucei* and *T. cruzi*, 7,8-dihydroxyflavone (pIC_50_ = 5.17), were selected for interpretation purposes. The dataset was based on values of pIC_50_ obtained from the literature for grouping flavonoids which present similar biological properties. It was observed that some flavonoids have similar clustering results associated with their pIC_50_ (Figure 4(b)).

A hierarchical clustering analysis, based on Euclidean distance, grouped fisetin and quercetin, chrysin and isorhamnetin, and 7-hydroxyflavone alone, due to its pIC_50_ (Figure 4(b)). Fisetin presented a strong-to-perfect correlation (*r* ≥ 0.99) to quercetin, chrysin, isorhamnetin, and 7-hydroxyflavone (Figure 4(c)), suggesting that these four flavonoids are similar to fisetin against *L. donovani*, *T. brucei*, *T. cruzi*, and L6 cell cytotoxicity. Besides, all the flavonoids of fisetin series (Figures 4(b) and 4(c)) have a difference in the logarithmic scale of at least one unity between activity and toxicity, indicating good therapeutic window. The lower correlation encountered in fisetin series was in kaempferol-3-*O*-rutinoside (*r* = 0.22) that is the less active flavonoid (pIC_50_: *L. donovani*, 4.3; *T. brucei*, 3.8; *T. cruzi*, 4.3; and L6 cells, 3.8).

The activities of flavonoids from the fisetin series (Figure 4(b)) are associated with important structural portions of a flavonoid for the effective biological activity against *L. donovani*. The flavonoid fisetin, which has the higher pIC_50_ against *L. donovani*, presents in its structure the C2=C3 + C4-C=O, C3-OH + C4-C=O, and the catechol group, in which the last two portions can generate free radicals participating also as prooxidant agents [15]. In the case of isorhamnetin, the absence of the catechol group decreased the pIC_50_ compared with that in fisetin, and the lack of both the catechol group and hydroxyls at carbons 3 and 5, as in the 7-hydroxiflavone, reduced even more the pIC_50_ of this compound. These findings corroborate the necessity of balance between antioxidant and prooxidant characteristics of flavonoids for the effective biological activity. The clustering analysis enforces that the structural moieties of these compounds investigated are associated with their behaviour on trypanosomatids. The compound kaempferol-3-*O*-rutinoside, which reached the lower correlation with fisetin, does not present the catechol group, and the presence of a glucoside portion is associated with lower activities against trypanosomatids [21].

The flavonoid 7,8-dihydroxyflavone was the most active compound against *T. brucei* (pIC_50_ = 6.57) and *T. cruzi* (pIC_50_ = 4.58), and its series revealed a strong correlation to the compounds (−)-epigallocatechin gallate, daidzein, (+)-catechin, and (−)-epicatechin (Figures 4(b) and 4(c)). The difference in fisetin series is the toxicity to L6 cells, which is higher in 7,8-dihydroxyflavone series. The compounds 7,8-dihydroxyflavone and (−)-epigallocatechin gallate presented elevated toxicity compared to pIC_50_ for *T. cruzi*. The compound kaempferol-3-*O*-rutinoside (with low activity—higher values of pIC_50_) reached the lower correlation value (*r* = 0.03) (Figure 4(c)). These results suggest that flavonoids from the 7,8-dihydroxyflavone series displayed similarity in the level of biological activity and lower values of correlation with kaempferol-3-*O*-rutinoside.

Tasdemir et al. [21] evaluated 69 flavonoids in vitro against trypanosomatids. They observed some important trends on chemical structures and biological activity. However, a good model to explain and predict antiprotozoal properties was not established. Thus, it was suggested that these compounds could not present SAR, be a heterogeneous group, or display a very low difference between the most and less active compounds, making them difficult to determine QSAR. The method used by Tasdemir was based on partial least square (PLS), which is a linear multivariate statistic. In the case of ANN, the method consists of nonlinear multivariate statistics, dealing with compounds that can act by different mechanisms of action. These nonlinear methods are considered a gold standard due to their high predictive ability in these cases [43]. Thus, we found an ANN able to predict antitrypanosomatid activities (*R*^2^ > 0.75, *Q*^2^ > 0.62, and *P*^2^ > 0.67; Table 2; Figure S1) and TEAC (Table 2, Figure S1), but not the cytotoxic activity in L6 cells (*R*^2^ = 0.63, *Q*^2^ < 0.5, and *P*^2^ < 0.5; Table 2; Figure S1). These findings suggest the existence of common chemical characteristic of flavonoids, which is responsible for their antitrypanosomatid activities. These features were determined by the statistical selection of descriptors with high values of merit (higher than 0.72) and the additional elimination of descriptors with redundant features. Values of *R*^2^ (goodness of fit) >0.6, *Q*^2^ (robustness), and *P*^2^ (predictive ability) must be both greater than 0.5 for a relevant prediction model [44–47]. In order to detect overfitting, the use of scrambling is essential. The scrambling is realized by the random mix of the experimental activity of all compounds. The resulting dataset must be evaluated as in the original training set. The proof that the predictive power of the model is achieved is given when the scrambling analysis results in values of *R*^2^ and *Q*^2^ lower than 0.5 [21, 44]. This is expected for a good predictive model because randomly mixing the experimental values of biological activity totally removes the relationship between structures and activity [44]. Thus, the scrambling test validated the ANN models with no overfitting (Table 2, Table S10). Besides, the values from external validation corroborate these findings, since *P*^2^ is higher than 0.59 and for scramble test lower than 0.16. This means that in vitro antitrypanosomatid activities and TEAC values of a novel or designed flavonoid can be predicted using these ANN models, since the models have *R*^2^ > 0.6, *Q*^2^, and *P*^2^ values higher than 0.59 and *R*^2^, *Q*^2^, and *P*^2^ values lower than 0.4 in the scramble test (Table 2, Table S10).

The descriptors used in the prediction models of activity against *T. brucei* (Table S4) were associated with atomic properties, such as atomic masses, polarizability, and electronegativity (Broto-Moreau autocorrelation, represented by ATSC6c); atomic properties taking into consideration the atom number and the topological distance (Moran coefficient, represented by MATS8s); and charge and polarizability (given by the ATSC6c, VR1-Dzp, and RDF55p). The values obtained by descriptors as MATS8s and E2s, which are associated with the intrinsic state, also present important influence on the QSAR model.

The descriptors that contribute to the QSAR model to predict activity against *T. cruzi* describe atomic properties accounting for the steric and electronic features of the molecule, such as Sanderson electronegativity, van der Waals volume, mass, polarizability, and Kier-Hall intrinsic state. The descriptors TDB4e, TDB9v, TDB4m, and Dp are autocorrelation descriptors calculated for 3D spatial molecular geometry, based on topological and geometric distances and electronegativity, van der Waals volume, mass, and polarizability, respectively. The descriptor C1SP2 is associated with the carbon type and doubly bound carbon bound to one other carbon. The descriptor SHBint5 is based on the sum of E-state. The E-state index combines the electronic state of the bonded atom within the molecule with its topological nature in the context of the whole molecular skeleton (Table S4) [48].

The model for *L. donovani* was best defined by descriptors which considered the first potential of ionization, van der Waals volume, and the polarizability calculated by ki and AATS7i, GATS8v, and BCUTp-1h descriptors, respectively. The topological distance based on the covalent radius (TDB1r) and the minimum atom-type H E-state (minHCsatu), which represent the H linked to sp^3^ carbons bonded to unsaturated carbon, was also important to describe the biological activity of the flavonoids against this parasite (Table S4).

The QSAR model for TEAC also contains descriptors which are associated with the molecular charges (MATS1c, BCUTc-1h), the first potential of ionization (ATSC3i), and polarizability (GATS3p). The other descriptors important for the TEAC model are associated with the I-state (VR2_Dzs) and valence path (AVP-1) (Table S4). These descriptors take into consideration important molecular characteristics for antioxidant activity of flavonoids.

Although the model built to predict the toxicity in L6 cells was not satisfactory for prediction purposes (*Q*^2^ and *P*^2^ < 0.5), it was well fitted (*R*^2^ > 0.6); thus, the physicochemical features described by its descriptors were also analysed. It was observed that the descriptors of flavonoids used in L6 cell toxicity ANN model (Table S4) are dissimilar to those of antitrypanosomatid activities, suggesting that the molecular characteristics for the activity and toxicity are different.

Analysing all these results, we can conclude that there is a relationship between particular physicochemical features of flavonoids, their trypanosomatid activities, and TEAC, corroborating previously discussed results. The geometry and the electronic effects on a molecule are influenced significantly by the position of OH groups in flavonoids; thus, they might be considered important in order to investigate the antioxidant activity as earlier reported [49] and also to investigate trypanosomatid activities.

The development of prediction models for prooxidant activity would be very important. However, this property did not exhibit a standardized protocol on the literature (Supplementary material, Table S3), which is essential to determine QSAR. The antioxidant values obtained from different literature data, performed through a standardized protocol, exhibited reproducible results even from different references (Supplementary material, Table S2). Furthermore, all antiprotozoal data used in this work were obtained from a standard protocol used by Tasdemir et al. [21].

Besides pro- and antioxidant properties, some flavonoids inhibit the cell cycle, inducing cell apoptosis in amastigotes and promastigotes of *L. donovani*. Flavonoids also inhibit the protein kinase, affecting the cellular proliferation, including that in epimastigote and amastigote forms of *T. cruzi* [8]. Thus, they can act as multitarget agents being promising compounds to the development of antitrypanosomal drugs, alone or in association with other active compounds, as in the case where the flavonoid quercetin was proven to be beneficial for the treatment of leishmaniasis when associated with the drug stibanate [20].

In this context, we can conclude that these models can be useful for the previous screening of flavonoid activity before in vitro experiments, a rational and advantageous strategy to save time to discover active compounds with potent antioxidant and/or antiparasitic activity against *L. donovani*, *T. cruzi*, and *T. brucei*.

## 4. Conclusions

Our major finding comprises the correlation between the chemical structures of the flavonoids and their antioxidant, prooxidant, and antitrypanosomatid activities. QSAR models based on nonlinear multivariate statistics (using ANN) were developed for the flavonoids according to their most important descriptors correlated to their biological activity against parasitic diseases. The values of *R*^2^, *Q*^2^, and *P*^2^ obtained in ANN models for trypanosomatids suggest that the methods have applicability domains to predict antitrypanosomatid and antioxidant activities of flavonoids. These models can be useful for helping the discovery of effective compounds that could be used in the treatments against *L. donovani*, *T. cruzi*, and *T. brucei* infections. Besides, it was discovered that flavonoids as fisetin, with double bond C2=C3, hydroxyl substituents, and other physicochemical features, including steric, electronic, and topological properties, have an important balance concerning both anti- and prooxidant activities. This balance indeed seems to be relevant for electing effective compounds for the treatment of trypanosomatid infections. The oxidant potential of flavonoids is dangerous to trypanosomatids, since they have a rudimentary antioxidant system compared to the host, while the antioxidant property can protect the host from damage caused by oxidative stress from his own immune response. Additionally, some flavonoids proved to act through other mechanisms of action, for example, inhibiting protein kinase. They could act in multiple protozoan targets with low side effects on the host, being promising compounds to the development of drugs, alone or in association with other drugs. Thus, the explanation about the multiple target property of these compounds in addition to important molecular characteristics is of summary connotation for the development of a robust method for these types of predictions.

## Supplementary Material

Supplementary session. Supplementary Table S1 – The substitution pattern of all the 40 flavonoids divided by classes. Supplementary Table S2 – TEAC values with more than one value from the literature. Supplementary Table S3 – Prooxidant values to some flavonoids. Supplementary Table S4 – PaDEL descriptors to each QSAR model, meaning and features. Supplementary Table S5 – PaDEL descriptors of flavonoids important to describe activity against L. donovani∗. Supplementary Table S6 – PaDEL descriptors of flavonoids important to describe activity against T. brucei∗. Supplementary Table S7 – PaDEL descriptors of flavonoids important to describe activity against T. cruzi∗. Supplementary Table S8 – PaDEL descriptors of flavonoids important to describe activity against L6 cells∗. Supplementary Table S9 – PaDEL descriptors of flavonoids important to describe antioxidant activity (TEAC)∗. Supplementary Table S10 – Results of ANNs external validation∗. Supplementary Table S11 – SMILES for each flavonoid used in this work. Supplementary Figure S1. Artificial Neural Networks (ANNs) built on Weka 3.8.0 using the classifier Multilayer Perceptron. They have applicability domain for prediction of IC50 of flavonoids in L. donovani (R2=0.75, Q2=0.62, P2=0.67), T. cruzi (R2=0.94, Q2=0.67, P2=0.92), T. brucei (R2=0.93, Q2=0.73, P2=0.66) and TEAC (R2=0.89, Q2=0.72, P2=0.66). All of them show R2, Q2 and P2 lower than 0.41 for scramble test. Except ANN built to predict toxicity against L6 cells that not show predictive ability even robustness (R2=0.63, Q2=0.33, P2=0.48).

## Figures and Tables

**Table 1:** Flavonoid series exhibiting the pIC_50_ values against *L. donovani*, *T. brucei*, *T. cruzi*, L6 cells, TEAC, and prooxidant activities reported.

Flavonoids	*L. donovani*	*T. brucei*	*T. cruzi*	L6 cells	TEAC^∗^	Ref. TEAC
(−)-Epicatechin	3.985	4.136	3.508	3.508	2.602	[50]
(−)-Epicatechin gallate	4.168	4.289	3.691	3.691	2.307	[12]
(−)-Epigallocatechin	4.009	4.884	3.579	4.321	2.420	[12]
(−)-Epigallocatechin gallate	4.380	4.692	3.707	4.494	2.323	[12]
(+)-Catechin	3.985	4.301	3.508	3.508	2.620	[50]
(+)-Taxifolin	4.006	4.319	4.006	3.529	2.721	[12]
3,6-Dihydroxyflavone	5.007	4.861	4.560	4.396	2.686	[51]
3,7-Dihydroxyflavone	4.886	5.174	4.451	3.888	2.783	[51]
3-Hydroxyflavone	5.517	5.663	4.475	4.203	2.975	[51]
3-Methoxyflavone	3.924	4.012	4.246	3.447	4.222	[51]
6-Hydroxyflavone	4.644	4.858	4.013	3.770	3.022	[51]
6-Methoxyflavone	3.924	3.933	4.109	3.447	5.000	[51]
7,8-Dihydroxyflavone	5.174	6.572	4.585	4.507	2.988	[52]
7-Hydroxyflavone	4.764	4.557	3.900	3.732	4.398	[51]
Apigenin	5.153	4.724	4.093	4.174	2.839	[12]
Biochanin A	5.055	4.948	4.152	3.635	2.936	[53]
Chrysin	5.063	4.681	4.091	3.893	2.845	[12]
Daidzein	3.928	4.665	3.451	3.451	2.903	[50]
Diosmetin	4.626	4.692	4.000	3.587	2.932	[54]
Eriodictyol	4.442	4.074	4.298	3.714	2.745	[12]
Fisetin	5.678	4.938	3.979	3.871	2.553	[51]
Flavone	4.648	4.540	3.935	3.720	3.523	[51]
Galangin	5.255	4.214	4.135	4.037	2.827	[12]
Genistein	4.528	5.318	4.062	4.111	2.538	[50]
Hesperidin	4.308	4.137	4.308	3.831	2.967	[50]
Hyperoside	4.189	4.301	4.189	3.712	2.633	[55]
Isorhamnetin	4.920	4.550	4.023	3.891	2.570	[55]
Kaempferol	4.994	4.493	4.078	3.882	2.873	[12]
Kaempferol-3-*O*-glucoside	4.342	3.806	4.174	3.697	3.223	[56]
Kaempferol-3-*O*-rutinoside	4.297	3.847	4.297	3.820	3.545	[56]
Luteolin	5.553	4.888	4.126	4.483	2.680	[12]
Luteolin-7-*O*-glucoside	5.610	3.869	3.697	3.697	2.833	[55]
Morin	5.033	3.897	4.003	3.526	2.585	[51]
Myricetin	5.389	4.301	4.025	3.938	2.509	[12]
Naringenin	4.736	3.771	3.958	3.480	2.824	[12]
Quercetin	5.480	4.561	4.003	3.911	2.328	[12]
Quercitrin	4.403	4.206	4.174	3.697	2.818	[54]
Rhamnetin	4.837	5.801	4.417	3.546	2.896	[55]
Rutin	4.308	4.161	4.308	3.831	2.620	[57]
Vitexin	4.158	3.890	4.158	3.681	3.666	[55]

^∗^TEAC for the same compounds evaluated in different research groups are reproducible. Data can be found at the supplementary session (Supplementary Table S2). The chemical structure of each flavonoid is represented in Supplementary Table S1, and its respective SMILES is provided in Supplementary Table S11.

**Table 2:** Values of *R*^2^, *Q*^2^, and *P*^2^ for the prediction model ANN using the descriptors of chemical features of the flavonoids statistically selected.

	*R* ^2^	*Q* ^2^	*P* ^2^	*R* ^2^ (scramble)	*Q* ^2^ (scramble)	*P* ^2^ (scramble)
*L. donovani*	0.75	0.62	0.67	0.22	0.04	0.16
*T. brucei*	0.93	0.73	0.67	0.15	0.09	0.01
*T. cruzi*	0.94	0.67	0.92	0.25	0.01	0.01
L6 cells	0.63	0.33	0.48	0.28	0.01	0.30
TEAC	0.89	0.72	0.66	0.41	0.05	0.09
