# Supplementary material for: The Correlation between Chemical Structures and Antioxidant, Prooxidant, and Antitrypanosomatid Properties of Flavonoids
Source: Oxid Med Cell Longev. 2017 Jul 2;2017:3789856. doi: 10.1155/2017/3789856 (PMC5511661; doi:10.1155/2017/3789856)
Supplement: Supplementary file 1 — Supplementary session. Supplementary Table S1 – The substitution pattern of all the 40 flavonoids divided by classes. Supplementary Table S2 – TEAC values with more than one value from the literature. Supplementary Table S3 – Prooxidant values to some flavonoids. Supplementary Table S4 – PaDEL descriptors to each QSAR model, meaning and features. Supplementary Table S5 – PaDEL descriptors of flavonoids important to describe activity against L. donovani∗. Supplementary Table S6 – PaDEL descriptors of flavonoids important to describe activity against T. brucei∗. Supplementary Table S7 – PaDEL descriptors of flavonoids important to describe activity against T. cruzi∗. Supplementary Table S8 – PaDEL descriptors of flavonoids important to describe activity against L6 cells∗. Supplementary Table S9 – PaDEL descriptors of flavonoids important to describe antioxidant activity (TEAC)∗. Supplementary Table S10 – Results of ANNs external validation∗. Supplementary Table S11 – SMILES for each flavonoid used in this work. Supplementary Figure S1. Artificial Neural Networks (ANNs) built on Weka 3.8.0 using the classifier Multilayer Perceptron. They have applicability domain for prediction of IC50 of flavonoids in L. donovani (R2=0.75, Q2=0.62, P2=0.67), T. cruzi (R2=0.94, Q2=0.67, P2=0.92), T. brucei (R2=0.93, Q2=0.73, P2=0.66) and TEAC (R2=0.89, Q2=0.72, P2=0.66). All of them show R2, Q2 and P2 lower than 0.41 for scramble test. Except ANN built to predict toxicity against L6 cells that not show predictive ability even robustness (R2=0.63, Q2=0.33, P2=0.48). [file 3789856.f1.docx]

**Supplementary Session**

**Supplementary Table S1 –** The substitution pattern of all the 40 flavonoids divided by classes.

|  | ***Flavan-3-ols*** | | | | | | | | | | | | | | | | |
| --- | --- | --- | --- | --- | --- | --- | --- | --- | --- | --- | --- | --- | --- | --- | --- | --- | --- |
|  |  | | | | | | | | | | | | | | | | |
|  | ***R_1_*** | | | | | ***R_2_*** | | | | | | | ***R_3_*** | | | | |
| *Catechin* | OH | | | | | OH | | | | | | | H | | | | |
| *Epicatechin* | OH | | | | | OH | | | | | | | H | | | | |
| *Epigallocatechin* | OH | | | | | OH | | | | | | | OH | | | | |
| *Epicatechingallate* | Gallate | | | | | OH | | | | | | | H | | | | |
| *Epigallocatechingallate* | Gallate | | | | | OH | | | | | | | OH | | | | |
|  | ***Flavanones*** | | | | | | | | | | | | | | | | |
|  |  | | | | | | | | | | | | | | | | |
|  | ***R_1_*** | | ***R_2_*** | | | | | | ***R_3_*** | | | | | ***R_4_*** | | | |
| *Taxifolin* | OH | | OH | | | | | | OH | | | | | OH | | | |
| *Eriodictyol* | H | | OH | | | | | | OH | | | | | OH | | | |
| *Narigenin* | H | | OH | | | | | | H | | | | | OH | | | |
| *Hesperidin* | H | | *O*-Rut | | | | | | OH | | | | | *O*-Me | | | |
|  | ***Isoflavones*** | | | | | | | | | | | | | | | | |
|  |  | | | | | | | | | | | | | | | | |
|  | ***R_1_*** | | | | | | | ***R_2_*** | | | | | | | | | |
| *Biochanin A* | OH | | | | | | | *O*-Me | | | | | | | | | |
| *Daidzein* | H | | | | | | | OH | | | | | | | | | |
| *Genistein* | OH | | | | | | | OH | | | | | | | | | |
|  | ***Flavones substituted only on the ring A and C*** | | | | | | | | | | | | | | | | |
|  |  | | | | | | | | | | | | | | | | |
|  | ***R_1_*** | | | | ***R_2_*** | | | | | | ***R_3_*** | | | | | ***R_4_*** | |
| *3,6-Dihydroxyflavone* | OH | | | | OH | | | | | | H | | | | | H | |
| *3,7-Dihydroxyflavone* | OH | | | | H | | | | | | OH | | | | | H | |
| *3-Hydroxyflavone* | OH | | | | H | | | | | | H | | | | | H | |
| *3-Methoxyflavone* | *O*-Me | | | | H | | | | | | H | | | | | H | |
| *6-Hydroxyflavone* | H | | | | OH | | | | | | H | | | | | H | |
| *6-Methoxyflavone* | H | | | | *O*-Me | | | | | | H | | | | | H | |
| *7,8-Dihydroxyflavone* | H | | | | H | | | | | | OH | | | | | OH | |
| *7-Hydroxyflavone* | H | | | | H | | | | | | OH | | | | | H | |
|  | ***Flavones substituted on the ring A, B, and C*** | | | | | | | | | | | | | | | | |
|  |  | | | | | | | | | | | | | | | | |
|  | ***R_1_*** | ***R_2_*** | | ***R_3_*** | | | ***R_4_*** | | | ***R_5_*** | | ***R_6_*** | | | ***R_7_*** | | ***R_8_*** |
| *Apigenin* | H | OH | | OH | | | H | | | H | | H | | | OH | | H |
| *Chrysin* | H | OH | | OH | | | H | | | H | | H | | | H | | H |
| *Diosmetin* | H | OH | | OH | | | H | | | H | | OH | | | *O*-Me | | H |
| *Fisetin* | OH | H | | OH | | | H | | | H | | OH | | | OH | | H |
| *Flavone* | H | H | | H | | | H | | | H | | H | | | H | | H |
| *Galangin* | OH | OH | | OH | | | H | | | H | | H | | | H | | H |
| *Hyperoside* | *O*-Gal | OH | | OH | | | H | | | H | | OH | | | OH | | H |
| *Isorhamnetin* | OH | OH | | OH | | | H | | | H | | *O*-Me | | | OH | | H |
| *Kaempferol* | OH | OH | | OH | | | H | | | H | | H | | | OH | | H |
| *Kaempferol-3-*O*-glucoside* | *O*-Glu | OH | | OH | | | H | | | H | | H | | | OH | | H |
| *Kaempferol-3-*O*-rutinoside* | *O*-Rut | OH | | OH | | | H | | | H | | H | | | OH | | H |
| *Luteolin* | H | OH | | OH | | | H | | | H | | OH | | | OH | | H |
| *Luteolin-7-*O*-glucoside* | H | OH | | *O*-Glu | | | H | | | H | | OH | | | OH | | H |
| *Morin* | OH | OH | | OH | | | H | | | OH | | H | | | OH | | H |
| *Myricetin* | OH | OH | | OH | | | H | | | H | | OH | | | OH | | OH |
| *Quercetin* | OH | OH | | OH | | | H | | | H | | OH | | | OH | | H |
| *Quercitrin* | *O*-Rha | OH | | OH | | | H | | | H | | OH | | | OH | | H |
| *Rhamnetin* | OH | OH | | *O*-Me | | | H | | | H | | OH | | | OH | | H |
| *Rutin* | *O*-Rut | OH | | OH | | | H | | | H | | OH | | | OH | | H |
| *Vitexin* | H | OH | | OH | | | Glu | | | H | | H | | | OH | | H |

**Supplementary Table S2** – TEAC values with more than one value from the literature.

| ***Flavonoids*** | ***TEAC 1*** | ***Reference*** | ***TEAC 2*** | ***Reference*** |
| --- | --- | --- | --- | --- |
| *6-Hydroxyflavone* | 0.95±0.18 | [40] | 0.95 | [44] |
| *Chrysin* | 1.43 | [12] | 2.52±0.12 | [40] |
| *Apigenin* | 1.45 | [12] | 2.80±0.50 | [40] |
| *Luteolin* | 2.09 | [12] | 2.48±0.23 | [40] |
| *Galangin* | 1.49 | [12] | 2.08±0.11 | [40] |
| *Kaempferol* | 1.34 | [12] | 1.45±0.08 | [40] |
| *Quercetin* | 4.7 | [12] | 4.84±0.45 | [40] |
| *Myricetin* | 3.1 | [12] | 3.08±0.46 | [40] |
| *Rutin* | 2.4±0. I2 | [46] | 2.4 | [12] |
| *Genistein* | 2.9 | [39] | 2.96±0.49 | [40] |

**Supplementary Table S3** – Prooxidant values to some flavonoids.

| ***FLAVONOIDS*** | ***L. donovani*** | ***T. brucei*** | ***T. cruzi*** | ***L6 cells*** | ***TEAC**** | ***Prooxidant*** | ***References*** |
| --- | --- | --- | --- | --- | --- | --- | --- |
| *Apigenin* | 5.153 | 4.724 | 4.093 | 4.174 | 2.839 | 69±7(k×10^3^ min^-1^) | [58] |
| *Naringenin* | 4.736 | 3.771 | 3.958 | 3.48 | 2.824 | 44±6 | [12] |
| *Rutin* | 4.308 | 4.161 | 4.308 | 3.831 | 2.62 | 4.1±0.4 (E^9^) ks (m^­1^.s^­1^) | [15] |
| *Fisetin* | 5.678 | 4.938 | 3.979 | 3.871 | 2.553 | 1841±121(k×10^3^ min^-1^) | [58] |
| *Morin* | 5.033 | 3.897 | 4.003 | 3.526 | 2.585 | 125-200μM | [59] |
| *Luteolin* | 5.553 | 4.888 | 4.126 | 4.483 | 2.68 | 1.54±0.14 | [15] |
| *(+)-Catechin* | 3.985 | 4.301 | 3.508 | 3.508 | 2.62 | 0.99 (no unity) | [60] |
| *(-)-Epicatechin* | 3.985 | 4.136 | 3.508 | 3.508 | 2.602 | 0.2-500 μM | [61] |
| *(+)-Taxifolin* | 4.006 | 4.319 | 4.006 | 3.529 | 2.721 | 0.2 (E^9^) ks (m^­1^.s^­1^) | [15] |
| *Eriodictyol* | 4.442 | 4.074 | 4.298 | 3.714 | 2.745 | 0.14 (E^9^) ks (m^­1^.s^­1^) | [15] |
| *Galangin* | 5.255 | 4.214 | 4.135 | 4.037 | 2.827 | 0.09 (μM quercetin/μg sample) | [62] |
| *3,7-Dihydroxyflavone* | 4.886 | 5.174 | 4.451 | 3.888 | 2.783 | 0.03 (μM quercetin/μg sample) | [62] |
| *(-)-Epigallocatechin* | 4.009 | 4.884 | 3.579 | 4.321 | 2.42 | 0.02-1000μM | [61] |
| *3,6-Dihydroxyflavone* | 5.007 | 4.861 | 4.56 | 4.396 | 2.686 | 0.01(μM quercetin/μg sample) | [62] |
| *3-Hydroxyflavone* | 5.517 | 5.663 | 4.475 | 4.203 | 2.975 | 0.01(μM quercetin/μg sample) | [62] |
| *7-Hydroxyflavone* | 4.764 | 4.557 | 3.9 | 3.732 | 4.398 | 0.00(μM quercetin/μg sample) | [62] |
| *Kaempferol* | 4.994 | 4.493 | 4.078 | 3.882 | 2.873 | 3* | [11] |
| *Quercetin* | 5.48 | 4.561 | 4.003 | 3.911 | 2.328 | 2* | [11] |
| *Myricetin* | 5.389 | 4.301 | 4.025 | 3.938 | 2.509 | 1* | [11] |

* The study of Cao et al. [11] carried out an experiment that represent well the *in vivo* system and did not represent the results as usual, the specific values and the unit were not provided. We established that the value 1 represent the most active and 5 the less active according the data from the article cited.

**Supplementary Table S4** – PaDEL descriptors to each QSAR model, meaning and features.

| ***PARASITE*** | ***DESCRIPTOR*** | ***DESCRIPTOR MEANING*** | ***FEATURE*** | ***REFERENCE*** |
| --- | --- | --- | --- | --- |
| ***L.donovani*** | Ki | K global shape index / weighted by relative first ionization potential | 3D | [56] |
|  | AATS7i | Average Broto-Moreau autocorrelation - lag 7 / weighted by first ionization potential | 2D | [63] |
|  | GATS8v | Geary autocorrelation - lag 8 / weighted by van der Waals volumes | 2D | [63] |
|  | BCUTp-1h | nlow highest polarizability weighted BCUTS | 2D | [64] |
|  | minHCsatu | Minimum atom-type H E-State: H on C sp3 bonded to unsaturated C | 2D | [56] |
|  | TDB1r | topological distance based autocorrelation - lag 1 / weighted by covalent radius | 3D | [63] |
| ***T. brucei*** | ATSC6c | Centered Broto-Moreau autocorrelation - lag 6 / weighted by charges | 2D | [63] |
|  | MATS8s | Moran autocorrelation - lag 8 / weighted by I-state | 2D | [63] |
|  | VR1_Dzp | Randic-like eigenvector-based index from Barysz matrix / weighted by polarizabilities | 2D | [63] |
|  | TDB6i | 3D topological distance based autocorrelation - lag 6 / weighted by first ionization potential | 3D | [63] |
|  | RDF55p | Radial distribution function - 055 / weighted by relative polarizabilities | 3D | [63] |
|  | E2s | 2nd component accessibility directional WHIM index / weighted by relative I-state | 3D | [63] |
| ***T. cruzi*** | C1SP2 | Doubly bound carbon bound to one other carbon | 2D | [63] |
|  | SHBint5 | Sum of E-State descriptors of strength for potential hydrogen bonds of path length 5 | 2D | [56] |
|  | TDB4m | 3D topological distance based autocorrelation - lag 4 / weighted by mass | 3D | [63] |
|  | TDB9v | 3D topological distance based autocorrelation - lag 9 / weighted by van der Waals volumes | 3D | [63] |
|  | TDB4e | 3D topological distance based autocorrelation - lag 4 / weighted by Sanderson electronegativities | 3D | [63] |
|  | Dp | D total accessibility index / weighted by relative polarizabilities | 3D | [63] |
| ***TEAC*** | ATSC3i | Centered Broto-Moreau autocorrelation - lag 3 / weighted by first ionization potential | 2D | [63] |
|  | MATS1c | Moran autocorrelation - lag 1 / weighted by charges | 2D | [63] |
|  | GATS3p | Geary autocorrelation - lag 3 / weighted by polarizabilities | 2D | [63] |
|  | VR2_Dzs | Normalized Randic-like eigenvector-based index from Barysz matrix / weighted by I-state | 2D | [63] |
|  | BCUTc-1h | nlow highest partial charge weighted BCUTS | 2D | [63] |
|  | AVP-1 | Average valence path, order 1 | 2D | [56] |
| ***L6 Cells*** | VR2_Dzv | Normalized Randic-like eigenvector-based index from Barysz matrix / weighted by van der Waals volumes | 2D | [63] |
|  | MDEC-11 | Molecular distance edge between all primary carbons | 2D | [65] |
|  | minHBint5 | Minimum E-State descriptors of strength for potential Hydrogen Bonds of path length 5 | 2D | [63] |
|  | maxHBint5 | Maximum E-State descriptors of strength for potential Hydrogen Bonds of path length 5 | 2D | [63] |
|  | CIC5 | Complementary information content index (neighborhood symmetry of 5-order) | 2D | [63] |

**Supplementary Table S5** – PaDEL descriptors of flavonoids important to describe activity against *L. donovani**

| ***FLAVONOIDS*** | ***AATS7i*** | ***GATS8v*** | ***BCUTp-1h*** | ***minHCsatu*** | ***TDB1r*** | ***Ki*** |
| --- | --- | --- | --- | --- | --- | --- |
| 2R3R-Epicatechin | 162.5659 | 1.076984 | 9.940203 | 0.713844 | 0.563377 | 0.61275 |
| 2R3R-Epicatechingallate | 156.0958 | 1.080316 | 10.70773 | 0.861063 | 0.575798 | 0.288641 |
| 2R3R-Epigallocatechin | 162.9996 | 1.027925 | 9.999018 | 0.734252 | 0.564358 | 0.644538 |
| 2R3R-Epigallocatechingallate | 156.528 | 1.066131 | 10.76655 | 0.881472 | 0.5764 | 0.280856 |
| 2R3S-Catechin | 162.5659 | 1.076984 | 9.940203 | 0.713844 | 0.563262 | 0.502046 |
| 3,6-Dihydroxyflavone | 156.6516 | 1.14147 | 9.943875 | 0 | 0.592224 | 0.691328 |
| 3,7-Dihydroxyflavone | 156.5709 | 1.291508 | 9.958578 | 0 | 0.592025 | 0.639445 |
| 3-Hydroxyflavone | 155.9808 | 1.2351 | 9.929173 | 0 | 0.592006 | 0.658002 |
| 3-Methoxyflavone | 156.7655 | 1.110763 | 10.24354 | 0 | 0.576762 | 0.5078 |
| 6-Hydroxyflavone | 153.5024 | 1.223016 | 9.696635 | 0 | 0.591873 | 0.717565 |
| 6-Methoxyflavone | 156.5449 | 1.104853 | 9.716284 | 0 | 0.576539 | 0.767569 |
| 7,8-Dihydroxyflavone | 154.3446 | 1.294757 | 9.770148 | 0 | 0.592139 | 0.636955 |
| 7-Hydroxyflavone | 153.5131 | 1.273769 | 9.711337 | 0 | 0.59177 | 0.663293 |
| Apigenin | 154.3562 | 1.236753 | 9.770155 | 0 | 0.591801 | 0.680736 |
| Bbiochanin_A | 153.754 | 1.271294 | 10.29814 | 0 | 0.577372 | 0.790597 |
| Chrysin | 153.5145 | 1.314542 | 9.740744 | 0 | 0.591758 | 0.643939 |
| Daidzein | 154.4305 | 1.116326 | 10.20003 | 0 | 0.591782 | 0.773868 |
| Diosmetin | 153.6047 | 1.205276 | 9.901216 | 0 | 0.578042 | 0.73924 |
| Eriodictyol | 159.4243 | 1.132904 | 9.785457 | 0.793039 | 0.578508 | 0.457195 |
| Fisetin | 157.6267 | 1.242254 | 10.0468 | 0 | 0.592321 | 0.63838 |
| Flavone | 152.5256 | 1.280858 | 9.681933 | 0 | 0.591714 | 0.686254 |
| Galangin | 157.1602 | 1.33325 | 9.987987 | 0 | 0.592035 | 0.62551 |
| Genistein | 155.1896 | 1.154803 | 10.25884 | 0 | 0.591811 | 0.748873 |
| Hesperidin | 160.521 | 1.007808 | 9.928756 | 0.854451 | 0.543237 | 0.774898 |
| Hyperoside | 155.0693 | 1.187992 | 10.88304 | 0 | 0.566112 | 0.386977 |
| Isorhamnetin | 159.2015 | 1.30514 | 10.15481 | 0 | 0.578637 | 0.572898 |
| Kaempferol | 157.709 | 1.311268 | 10.02497 | 0 | 0.592068 | 0.657478 |
| Kaempferol-3-O-glucoside | 154.8538 | 1.178096 | 10.82423 | 0 | 0.565228 | 0.38897 |
| Kaempferol-3-O-rutinoside | 158.6052 | 1.093621 | 10.84923 | 0 | 0.552673 | 0.190026 |
| Luteolin | 155.0587 | 1.246418 | 9.829109 | 0 | 0.592046 | 0.681169 |
| Luteolin-7-O-glucoside | 157.1411 | 1.059373 | 9.929837 | 0 | 0.565063 | 0.685983 |
| Morin | 158.6522 | 1.278006 | 10.13503 | 0 | 0.592107 | 0.638944 |
| Myricetin | 158.465 | 1.271305 | 10.13503 | 0 | 0.592523 | 0.665579 |
| Naringenin | 158.5358 | 1.121786 | 9.726643 | 0.765261 | 0.57786 | 0.642421 |
| Quercetin | 158.1106 | 1.287107 | 10.07621 | 0 | 0.592283 | 0.654396 |
| Quercitrin | 154.3607 | 1.198684 | 10.87569 | 0 | 0.564796 | 0.389516 |
| Rhamnetin | 157.514 | 1.254603 | 10.1155 | 0 | 0.578605 | 0.576893 |
| Rutin | 158.6867 | 1.101701 | 10.90803 | 0 | 0.55334 | 0.531907 |
| Taxifolin | 161.5321 | 1.171722 | 10.0207 | 1.035625 | 0.579655 | 0.408691 |
| Vitexin | 158.5572 | 1.153625 | 11.19703 | 1.115409 | 0.566711 | 0.376351 |

* Descriptors were selected based on the original IC_50_ values according to Tasdemir et al [21].

**Supplementary Table S6** – PaDEL descriptors of flavonoids important to describe activity against *T. brucei**

| **Name** | **ATSC6c** | **MATS8s** | **VR1_Dzp** | **TDB6i** | **RDF55p** | **E2s** |
| --- | --- | --- | --- | --- | --- | --- |
| 2R3R-Epicatechin | -0.04133 | -0.36561 | 245.2217 | 912.3286 | 1.781458 | 0.456198 |
| 2R3R-Epicatechingallate | 0.046434 | -0.18652 | 241.4115 | 879.5999 | 16.57869 | 0.477452 |
| 2R3R-Epigallocatechin | 0.120004 | -0.3612 | 221.2932 | 926.188 | 2.910269 | 0.458972 |
| 2R3R-Epigallocatechingallate | 0.182299 | -0.14554 | 270.1438 | 882.8468 | 12.4049 | 0.508056 |
| 2R3S-Catechin | -0.04133 | -0.36561 | 157.3727 | 889.1378 | 5.804695 | 0.485179 |
| 3,6-Dihydroxyflavone | -0.0217 | 0.098851 | 195.1102 | 910.6803 | 2.524366 | 0.495729 |
| 3,7-Dihydroxyflavone | 0.039902 | -0.19619 | 165.5086 | 906.7452 | 2.645628 | 0.505524 |
| 3-Hydroxyflavone | -0.03797 | 0.03621 | 176.168 | 903.1638 | 2.484446 | 0.505394 |
| 3-Methoxyflavone | -0.04085 | 0.07144 | 135.4331 | 874.9909 | 4.034434 | 0.451181 |
| 6-Hydroxyflavone | -0.19764 | 0.029928 | 144.3844 | 926.6672 | 1.467106 | 0.495859 |
| 6-Methoxyflavone | -0.03799 | 0.032754 | 697.2502 | 923.1548 | 2.173732 | 0.471703 |
| 7,8-Dihydroxyflavone | 0.143167 | -0.18248 | 224.7257 | 922.1844 | 1.629292 | 0.507137 |
| 7-Hydroxyflavone | 0.171571 | -0.13205 | 166.2614 | 922.5833 | 1.508527 | 0.504751 |
| Apigenin | 0.435976 | 0.07274 | 160.145 | 925.9509 | 1.81365 | 0.48769 |
| Bbiochanin_A | 0.040191 | -0.02106 | 188.0406 | 941.1867 | 3.105136 | 0.484135 |
| Chrysin | 0.253865 | -0.22505 | 310.0187 | 924.6541 | 1.990447 | 0.497449 |
| Daidzein | -0.06161 | 0.024012 | 159.9594 | 937.8348 | 1.775017 | 0.511667 |
| Diosmetin | 0.25841 | -0.2415 | 176.5529 | 930.2389 | 1.753051 | 0.493511 |
| Eriodictyol | 0.175294 | -0.07233 | 199.4226 | 888.7839 | 6.673435 | 0.522827 |
| Fisetin | 0.12127 | -0.13729 | 251.2039 | 914.452 | 2.535822 | 0.511189 |
| Flavone | 0.001569 | -0.06148 | 141.3026 | 919.1036 | 0.934728 | 0.522654 |
| Galangin | -0.05193 | -0.27739 | 215.684 | 911.0768 | 2.948528 | 0.494667 |
| Genistein | 0.029671 | -0.08983 | 183.5445 | 937.6917 | 2.650288 | 0.49722 |
| Hesperidin | 0.229329 | -0.03008 | 573.4996 | 888.3705 | 15.70739 | 0.411703 |
| Hyperoside | 0.087867 | -0.00731 | 298.9593 | 858.9495 | 12.63083 | 0.414615 |
| Isorhamnetin | 0.110467 | -0.23105 | 244.972 | 905.0814 | 3.407144 | 0.529041 |
| Kaempferol | 0.037413 | -0.1371 | 168.6892 | 912.7259 | 3.615411 | 0.466541 |
| Kaempferol-3-O-glucoside | 0.112345 | 0.046482 | 321.6571 | 839.2009 | 16.24366 | 0.424823 |
| Kaempferol-3-O-rutinoside | 0.134988 | 0.020073 | 546.9923 | 846.2231 | 30.77049 | 0.401679 |
| Luteolin | 0.220578 | -0.11206 | 177.8852 | 932.7285 | 2.070341 | 0.510534 |
| Luteolin-7-O-glucoside | 0.215071 | -0.06939 | 758.3307 | 854.3598 | 8.026986 | 0.439984 |
| Morin | 0.096584 | 0.164632 | 166.8919 | 906.1004 | 3.898384 | 0.508562 |
| Myricetin | 0.087061 | -0.34524 | 186.3304 | 929.5997 | 2.851669 | 0.513738 |
| Naringenin | 0.369769 | 0.102779 | 153.8345 | 926.8107 | 3.387434 | 0.400398 |
| Quercetin | 0.037868 | -0.25702 | 225.4637 | 919.0761 | 3.8489 | 0.477449 |
| Quercitrin | 0.095053 | -0.0685 | 261.9495 | 862.8842 | 13.18725 | 0.418898 |
| Rhamnetin | -0.15625 | -0.21571 | 236.1777 | 923.2949 | 3.59776 | 0.438202 |
| Rutin | 0.110512 | -0.01256 | 624.9093 | 850.6337 | 23.82811 | 0.510966 |
| Taxifolin | 0.011231 | -0.21254 | 177.0744 | 907.1757 | 7.427398 | 0.470235 |
| Vitexin | 0.494542 | -0.06543 | 543.4783 | 892.3866 | 7.926201 | 0.415819 |

* Descriptors were selected based on the original IC_50_ values according to Tasdemir et al [21].

**Supplementary Table S7** – PaDEL descriptors of flavonoids important to describe activity against *T. cruzi**

| **Name** | **C1SP2** | **SHBint5** | **TDB4m** | **TDB9v** | **TDB4e** | **Dp** |
| --- | --- | --- | --- | --- | --- | --- |
| 2R3R-Epicatechin | 0 | 12.47437 | 275.3286 | 1137.068 | 31.71667 | 0.961715 |
| 2R3R-Epicatechingallate | 1 | 26.14776 | 313.2906 | 1264.136 | 32.78726 | 1.17843 |
| 2R3R-Epigallocatechin | 0 | 15.61785 | 294.5046 | 1247.98 | 32.11423 | 0.962549 |
| 2R3R-Epigallocatechingallate | 1 | 29.083 | 324.7858 | 1275.741 | 32.99886 | 1.163366 |
| 2R3S-Catechin | 0 | 12.47437 | 273.9285 | 1012.263 | 31.76163 | 0.937585 |
| 3,6-Dihydroxyflavone | 0 | 10.76575 | 352.9876 | 1152.804 | 33.67175 | 0.908132 |
| 3,7-Dihydroxyflavone | 0 | 0 | 357.1172 | 937.2301 | 33.74251 | 0.911175 |
| 3-Hydroxyflavone | 0 | 0 | 346.2321 | 887.8816 | 33.5485 | 0.939283 |
| 3-Methoxyflavone | 0 | 0 | 355.3089 | 711.2994 | 33.00732 | 0.877725 |
| 6-Hydroxyflavone | 0 | 10.77778 | 322.2857 | 1152.572 | 32.95569 | 0.957628 |
| 6-Methoxyflavone | 0 | 0 | 315.6651 | 1412.627 | 32.5343 | 0.911482 |
| 7,8-Dihydroxyflavone | 0 | 6.759394 | 349.7444 | 906.9774 | 33.83859 | 0.929761 |
| 7-Hydroxyflavone | 0 | 0 | 326.7174 | 969.3305 | 33.0354 | 0.956197 |
| Apigenin | 0 | 0 | 372.057 | 1158.527 | 33.86348 | 0.916606 |
| Bbiochanin_A | 1 | 0 | 360.7521 | 1245.842 | 33.26973 | 0.916093 |
| Chrysin | 0 | 0 | 380.4205 | 1018.592 | 33.80459 | 0.940806 |
| Daidzein | 1 | 0 | 320.6888 | 1378.217 | 32.98933 | 0.966494 |
| Diosmetin | 0 | 3.079023 | 377.4685 | 1345.883 | 33.79052 | 0.833816 |
| Eriodictyol | 0 | 3.24416 | 322.6829 | 1032.194 | 32.98807 | 0.998931 |
| Fisetin | 0 | 3.232759 | 357.4576 | 1148.894 | 34.16494 | 0.936362 |
| Flavone | 0 | 0 | 312.7427 | 887.0934 | 32.77065 | 1.007015 |
| Galangin | 0 | 10.84591 | 405.8959 | 991.5261 | 34.43002 | 0.907153 |
| Genistein | 1 | 0 | 370.0466 | 1303.104 | 33.69901 | 0.911352 |
| Hesperidin | 0 | 9.078199 | 275.5702 | 1315.476 | 32.08516 | 1.019542 |
| Hyperoside | 0 | 20.57294 | 344.2642 | 1255.717 | 33.17464 | 0.926876 |
| Isorhamnetin | 0 | 10.71888 | 398.4932 | 1156.808 | 34.28652 | 1.081469 |
| Kaempferol | 0 | 10.85156 | 396.5434 | 1095.647 | 34.45443 | 0.863129 |
| Kaempferol-3-O-glucoside | 0 | 17.62974 | 329.4145 | 1167.321 | 33.01624 | 0.966571 |
| Kaempferol-3-O-rutinoside | 0 | 8.738206 | 296.7704 | 1116.472 | 32.48643 | 1.086072 |
| Luteolin | 0 | 3.25659 | 378.0292 | 1276.111 | 34.21554 | 0.949436 |
| Luteolin-7-O-glucoside | 0 | 18.34584 | 313.3588 | 1518.567 | 32.71757 | 0.988367 |
| Morin | 0 | 21.54195 | 422.8365 | 1077.966 | 34.95547 | 0.92976 |
| Myricetin | 0 | 17.17102 | 418.2744 | 1297.424 | 35.08463 | 0.958653 |
| Naringenin | 0 | 0 | 323.3151 | 1147.603 | 32.75308 | 0.866055 |
| Quercetin | 0 | 14.04468 | 400.7823 | 1206.8 | 34.75063 | 0.881465 |
| Quercitrin | 0 | 8.206395 | 331.5911 | 1242.071 | 33.13225 | 0.890021 |
| Rhamnetin | 0 | 13.84468 | 389.9311 | 1299.953 | 34.28618 | 0.881273 |
| Rutin | 0 | 11.57789 | 294.6423 | 1137.028 | 32.3952 | 1.006973 |
| Taxifolin | 0 | 13.05577 | 344.7372 | 983.2711 | 33.48254 | 0.956155 |
| Vitexin | 0 | 18.77858 | 335.4892 | 1189.797 | 32.89853 | 0.997088 |

* Descriptors were selected based on the original IC_50_ values according to Tasdemir et al [21].

**Supplementary Table S8** – PaDEL descriptors of flavonoids important to describe activity against L6 cells*

| **Name** | **VR2_Dzv** | **minHBint5** | **maxHBint5** | **CIC5** | **MDEC-11** |
| --- | --- | --- | --- | --- | --- |
| 2R3R-Epicatechin | 10.02709 | 3.200831 | 5.365483 | 0.057143 | 0 |
| 2R3R-Epicatechingallate | 8.002522 | 2.562874 | 7.160541 | 0.12 | 0 |
| 2R3R-Epigallocatechin | 9.725008 | 3.168897 | 5.370363 | 0.166667 | 0 |
| 2R3R-Epigallocatechingallate | 8.536843 | 2.543488 | 7.170435 | 0.235294 | 0 |
| 2R3S-Catechin | 10.74608 | 3.200831 | 5.365483 | 0.057143 | 0 |
| 3,6-Dihydroxyflavone | 9.988526 | 3.395601 | 7.370147 | 0.137931 | 0 |
| 3,7-Dihydroxyflavone | 8.090239 | 0 | 0 | 0.137931 | 0 |
| 3-Hydroxyflavone | 9.076789 | 0 | 0 | 0.214286 | 0 |
| 3-Methoxyflavone | 7.754331 | 0 | 0 | 0.346932 | 0 |
| 6-Hydroxyflavone | 7.86138 | 3.468494 | 7.309285 | 0.214286 | 0 |
| 6-Methoxyflavone | 11.07014 | 0 | 0 | 0.282416 | 0 |
| 7,8-Dihydroxyflavone | 9.010332 | 6.759394 | 6.759394 | 0.137931 | 0 |
| 7-Hydroxyflavone | 8.145511 | 0 | 0 | 0.142857 | 0 |
| Apigenin | 7.873637 | 0 | 0 | 0.2 | 0 |
| Bbiochanin_A | 11.06023 | 0 | 0 | 0.204694 | 0 |
| Chrysin | 10.26422 | 0 | 0 | 0.137931 | 0 |
| Daidzein | 8.877929 | 0 | 0 | 0.137931 | 0 |
| Diosmetin | 10.67473 | 3.079023 | 3.079023 | 0.13985 | 0 |
| Eriodictyol | 8.912479 | 3.24416 | 3.24416 | 0.060606 | 0 |
| Fisetin | 11.43422 | 3.232759 | 3.232759 | -8.88E-16 | 0 |
| Flavone | 7.872386 | 0 | 0 | 0.296296 | 0 |
| Galangin | 9.618038 | 5.170914 | 5.674991 | 0.133333 | 0 |
| Genistein | 8.907416 | 0 | 0 | 0.133333 | 0 |
| Hesperidin | 12.64901 | 1.861348 | 2.868287 | 0.2274 | 0.05 |
| Hyperoside | 8.270604 | 2.447417 | 5.527323 | 0.037736 | 0 |
| Isorhamnetin | 31.00023 | 5.053486 | 5.665394 | 0.135854 | 0 |
| Kaempferol | 8.167755 | 5.172693 | 5.678866 | 0.129032 | 0 |
| Kaempferol-3-O-glucoside | 8.493798 | 2.465933 | 5.525492 | 0.115385 | 0 |
| Kaempferol-3-O-rutinoside | 13.21382 | 1.808002 | 2.614884 | 0.204929 | 0 |
| Luteolin | 8.64437 | 3.25659 | 3.25659 | -8.88E-16 | 0 |
| Luteolin-7-O-glucoside | 17.96637 | 2.503903 | 5.597966 | 0.038462 | 0 |
| Morin | 8.075992 | 5.175473 | 5.686461 | 0 | 0 |
| Myricetin | 10.17908 | 3.152257 | 5.689415 | 0.181818 | 0 |
| Naringenin | 8.273698 | 0 | 0 | 0.1875 | 0 |
| Quercetin | 11.70022 | 3.185648 | 5.684141 | 0 | 0 |
| Quercitrin | 8.313472 | 2.46934 | 3.014337 | 0.09144 | 0 |
| Rhamnetin | 9.180852 | 3.22454 | 5.45487 | 0.135854 | 0 |
| Rutin | 14.05648 | 1.803158 | 2.884141 | 0.147327 | 0 |
| Taxifolin | 11.32217 | 3.164532 | 5.581578 | 1.78E-15 | 0 |
| Vitexin | 15.83305 | 1.992101 | 5.352532 | 0.117647 | 0 |

* Descriptors were selected based on the original IC_50_ values according to Tasdemir et al [21].

**Supplementary Table S9** – PaDEL descriptors of flavonoids important to describe antioxidant activity (TEAC)*

| **Name** | **ATSC3i** | **MATS1c** | **GATS3p** | **VR2_Dzs** | **BCUTc-1h** | **AVP-1** |
| --- | --- | --- | --- | --- | --- | --- |
| 2R3R-Epicatechin | -17.7806 | -0.51051 | 1.158534 | 11.67732 | 0.195022 | 0.276913 |
| 2R3R-Epicatechingallate | -31.3497 | -0.54033 | 1.204433 | 12.44402 | 0.293895 | 0.266579 |
| 2R3R-Epigallocatechin | -19.5136 | -0.5162 | 1.188425 | 11.25272 | 0.269212 | 0.27122 |
| 2R3R-Epigallocatechingallate | -33.0265 | -0.54122 | 1.224441 | 11.54939 | 0.293971 | 0.263071 |
| 2R3S-Catechin | -17.7806 | -0.51051 | 1.158534 | 9.451187 | 0.127231 | 0.276913 |
| 3,6-Dihydroxyflavone | -10.2813 | -0.42593 | 1.055319 | 12.74191 | 0.220768 | 0.271824 |
| 3,7-Dihydroxyflavone | -10.2813 | -0.42382 | 1.055319 | 11.06289 | 0.22069 | 0.271824 |
| 3-Hydroxyflavone | -8.04199 | -0.37434 | 1.026022 | 8.071893 | 0.220678 | 0.278701 |
| 3-Methoxyflavone | -6.32552 | -0.26891 | 1.001361 | 26.32054 | 0.220917 | 0.283942 |
| 6-Hydroxyflavone | -8.04276 | -0.41479 | 1.028466 | 9.635666 | 0.19093 | 0.278102 |
| 6-Methoxyflavone | -6.32629 | -0.30757 | 1.00367 | 11.10982 | 0.19093 | 0.283372 |
| 7,8-Dihydroxyflavone | -10.2813 | -0.44711 | 1.055319 | 9.214936 | 0.190979 | 0.271824 |
| 7-Hydroxyflavone | -8.04276 | -0.41157 | 1.028466 | 8.941947 | 0.190841 | 0.278102 |
| Apigenin | -12.4007 | -0.46426 | 1.078715 | 10.18057 | 0.193519 | 0.2653 |
| Bbiochanin_A | -12.622 | -0.37888 | 1.084638 | 9.004819 | 0.200638 | 0.27036 |
| Chrysin | -10.3279 | -0.42322 | 1.048833 | 8.932369 | 0.193515 | 0.271539 |
| Daidzein | -13.3266 | -0.43033 | 1.089455 | 9.099863 | 0.225939 | 0.270916 |
| Diosmetin | -11.8116 | -0.44186 | 1.08414 | 15.74108 | 0.193541 | 0.265235 |
| Eriodictyol | -15.9878 | -0.46936 | 1.127992 | 8.316821 | 0.170873 | 0.270939 |
| Fisetin | -14.2776 | -0.48419 | 1.114077 | 20.53073 | 0.220697 | 0.260125 |
| Flavone | -5.61628 | -0.32289 | 0.99978 | 8.08681 | 0.190828 | 0.285671 |
| Galangin | -12.3999 | -0.42817 | 1.076367 | 9.341394 | 0.223258 | 0.265844 |
| Genistein | -15.1569 | -0.43492 | 1.109863 | 10.72926 | 0.240822 | 0.264978 |
| Hesperidin | -6.7233 | -0.4413 | 1.057212 | 21.05061 | 0.235691 | 0.287345 |
| Hyperoside | -5.6475 | -0.46241 | 1.039439 | 10.35081 | 0.223743 | 0.267866 |
| Isorhamnetin | -13.408 | -0.44277 | 1.112277 | 15.77661 | 0.223271 | 0.260476 |
| Kaempferol | -14.3238 | -0.4605 | 1.106668 | 11.73993 | 0.223258 | 0.260125 |
| Kaempferol-3-O-glucoside | -3.52337 | -0.45111 | 1.017385 | 10.31869 | 0.223732 | 0.271511 |
| Kaempferol-3-O-rutinoside | -6.2506 | -0.4525 | 1.037654 | 15.90385 | 0.223851 | 0.279733 |
| Luteolin | -14.3242 | -0.48538 | 1.107822 | 12.44486 | 0.1949 | 0.259865 |
| Luteolin-7-O-glucoside | -3.52375 | -0.46692 | 1.018005 | 14.18142 | 0.235665 | 0.27134 |
| Morin | -16.1602 | -0.4696 | 1.128858 | 29.56265 | 0.223276 | 0.255131 |
| Myricetin | -17.7839 | -0.48696 | 1.165942 | 23.24322 | 0.269426 | 0.250537 |
| Naringenin | -14.0392 | -0.44325 | 1.098641 | 7.589567 | 0.170832 | 0.276878 |
| Quercetin | -16.1139 | -0.4787 | 1.136151 | 54.51739 | 0.252289 | 0.255131 |
| Quercitrin | -6.63266 | -0.46747 | 1.038162 | 10.36219 | 0.223735 | 0.271316 |
| Rhamnetin | -13.408 | -0.44287 | 1.112277 | 10.28384 | 0.223271 | 0.260476 |
| Rutin | -8.3777 | -0.4609 | 1.054525 | 15.4766 | 0.223869 | 0.276766 |
| Taxifolin | -16.7226 | -0.46424 | 1.142658 | 8.157851 | 0.201362 | 0.264583 |
| Vitexin | -4.70834 | -0.44373 | 1.030877 | 12.44242 | 0.193639 | 0.275051 |

* Descriptors were selected based on the original IC_50_ values according to Tasdemir et al [21].

**Supplementary Table S10 –** Results of ANNs external validation*

|  | ***T. brucei*** | | | ***T. cruzi*** | | | ***TEAC*** | | |
| --- | --- | --- | --- | --- | --- | --- | --- | --- | --- |
| ***Flavonoid*** | ***IC_50_ real*** | ***predicted*** | ***error*** | ***IC_50_ real*** | ***predicted*** | ***error*** | ***IC_50_ real*** | ***predicted*** | ***error*** |
| 2R-3R-Epicatechin | 21.2 | 5.427 | -15.773 | 90 | 92.704 | 2.704 | 2.5 | 3.003 | 0.503 |
| Kaempferol-3-*O*-rutinoside | 84.5 | 69.095 | -15.405 | 30 | 15.885 | -14.115 | 0.285 | 1.738 | 1.453 |
| 3,6-Dihydroxyflavonde | 3.5 | 14.3 | 10.8 | 7 | 21.181 | 14.181 | 2.06 | 1.644 | -0.416 |
| Fisetin | 3.3 | 6.468 | 3.168 | 30 | 20.728 | -9.272 | 2.8 | 2.581 | -0.219 |
| 3-methoxyflavone | 24.5 | 21.744 | -2.756 | 14.3 | -0.192 | -14.492 | -0.01 | 0.047 | 0.057 |
| Luteolin-7-*O*-glucoside | 60.6 | 27.746 | -32.854 | 90 | 88.394 | -1.606 | 1.47 | 1.76 | 0.29 |
| Vitexin | 55.7 | 45.365 | -10.335 | 30 | 17.776 | -12.224 | 0.216 | 1.128 | 0.912 |
| 6-methoxyflavone | 29.4 | 8.406 | -20.994 | 19.6 | 18.677 | -0.923 | -0.04 | 0.133 | 0.173 |
| Quercetin | 8.3 | 5.577 | -2.723 | 30 | 21.728 | -8.272 | 4.7 | 2.96 | -1.74 |
| Kaempferol | 9.2 | 6.354 | -2.846 | 23.9 | 20.357 | -3.543 | 1.34 | 2.322 | 0.982 |
| 7,8-Dihydroxyflavone | 0.068 | 5.972 | 5.904 | 6.6 | 5.721 | -0.879 | 1.028 | 1.535 | 0.507 |
| Diosmetin | 6.1 | 5.895 | -0.205 | 30 | 21.377 | -8.623 | 1.17 | 1.574 | 0.404 |
| Apigenin | 5.1 | 43.121 | 38.021 | 21.8 | 21.273 | -0.527 | 1.45 | 1.885 | 0.435 |
| Isorhamnetin | 8.9 | 5.778 | -3.122 | 30 | 19.813 | -10.187 | 2.69 | 2.038 | -0.652 |

|  | ***L. donovani*** | | | ***L6 cells*** | | |
| --- | --- | --- | --- | --- | --- | --- |
| ***Flavonoid*** | ***IC_50_ real*** | ***predicted*** | ***error*** | ***IC_50_ real*** | ***predicted*** | ***error*** |
| 2R-3R-Epicatechin | 30 | 29.161 | -0.839 | 90 | 87.814 | -2.186 |
| Kaempferol-3-*O*-rutinoside | 30 | 29.698 | -0.302 | 37.5 | 43.153 | 5.653 |
| 3,6-Dihydroxyflavonde | 2.5 | 2.978 | 0.478 | 90 | 94.007 | 4.007 |
| Fisetin | 0.6 | 2.851 | 2.251 | 55.6 | 83.511 | 27.911 |
| 3-methoxyflavone | 30 | 26.351 | -3.649 | 14.4 | 36.863 | 22.463 |
| Luteolin-7-*O*-glucoside | 1.1 | 9.502 | 8.402 | 90 | 88.712 | -1.288 |
| Vitexin | 30 | 29.698 | -0.302 | 90 | 88.737 | -1.263 |
| 6-methoxyflavone | 30 | 2.86 | -27.14 | 40.4 | 37.632 | -2.768 |
| Quercetin | 1 | 2.851 | 1.851 | 90 | 90.374 | 0.374 |
| Kaempferol | 2.9 | 2.851 | -0.049 | 90 | 58.348 | -31.652 |
| 7,8-Dihydroxyflavone | 1.7 | 2.851 | 1.151 | 90 | 67.189 | -22.811 |
| Diosmetin | 7.1 | 2.851 | -4.249 | 90 | 39.128 | -50.872 |
| Apigenin | 1.9 | 2.851 | 0.951 | 44.1 | 20.475 | -23.625 |
| Isorhamnetin | 3.8 | 2.851 | -0.949 | 90 | 88.695 | -1.305 |

*The training set (26 flavonoids) was randomly selected by Weka 3.8 and the remainder 14 flavonoids were used as test set.

**Supplementary Table S11 –** SMILES for each flavonoid used in this work

| **FLAVONOID** | **SMILES** |
| --- | --- |
| ***2R3R-Epicatechin*** | OC1=CC(O)=C2C(O[C@@](C3=CC(O)=C(O)C=C3)([C@@H](O)C2)[H])=C1 |
| ***2R3R-Epicatechingallate*** | O=C(C1=CC(O)=C(O)C(O)=C1)O[C@@H]2CC3=C(O[C@@H]2C4=CC(O)=C(O)C=C4)C=C(O)C=C3O |
| ***2R3R-Epigallocatechin*** | O[C@@H]1[C@H](C(C=C2O)=CC(O)=C2O)OC3=CC(O)=CC(O)=C3C1 |
| ***2R3R-Epigallocatechingallate*** | OC1=C2C[C@@H]([C@H](C(C=C3O)=CC(O)=C3O)OC2=CC(O)=C1)OC(C(C=C4O)=CC(O)=C4O)=O |
| ***2R3S-Catechin*** | O[C@H]1[C@H](C(C=C2)=CC(O)=C2O)OC3=CC(O)=CC(O)=C3C1 |
| ***3,6-Dihydroxyflavone*** | OC1=C(C2=CC=CC=C2)OC(C=C3)=C(C=C3O)C1=O |
| ***3,7-Dihydroxyflavone*** | O=C1C(O)=C(OC2=CC(O)=CC=C12)C3=CC=CC=C3 |
| ***3-Hydroxyflavone*** | O=C1C(O)=C(C2=CC=CC=C2)OC3=CC=CC=C13 |
| ***3-Methoxyflavone*** | O=C1C2=CC=CC=C2OC(C3=CC=CC=C3)=C1OC |
| ***6-Hydroxyflavone*** | O=C1C(C=C2O)=C(C=C2)OC(C3=CC=CC=C3)=C1 |
| ***6-Methoxyflavone*** | O=C1C(C=C2OC)=C(C=C2)OC(C3=CC=CC=C3)=C1 |
| ***7,8-Dihydroxyflavone*** | OC1=C(O)C(OC(C2=CC=CC=C2)=CC3=O)=C3C=C1 |
| ***7-Hydroxyflavone*** | OC1=CC(OC(C2=CC=CC=C2)=CC3=O)=C3C=C1 |
| ***Apigenin*** | OC1=CC=C(C2=CC(C3=C(C=C(C=C3O)O)O2)=O)C=C1 |
| ***Bbiochanin_A*** | OC1=CC(O)=CC2=C1C(C(C3=CC=C(OC)C=C3)=CO2)=O |
| ***Chrysin*** | OC1=CC(O)=C2C(C=C(C3=CC=CC=C3)OC2=C1)=O |
| ***Daidzein*** | O=C1C(C2=CC=C(C=C2)O)=COC3=CC(O)=CC=C13 |
| ***Diosmetin*** | COC1=CC=C(C2=CC(C3=C(O)C=C(O)C=C3O2)=O)C=C1O |
| ***Eriodictyol*** | O=C1C(C(O)=CC(O)=C2)=C2O[C@@H](C1)C(C=C3)=CC(O)=C3O |
| ***Fisetin*** | OC1=CC(OC(C2=CC(O)=C(O)C=C2)=C(O)C3=O)=C3C=C1 |
| ***Flavone*** | O=C1C=C(C2=CC=CC=C2)OC3=CC=CC=C13 |
| ***Galangin*** | OC1=CC(OC(C2=CC=CC=C2)=C(O)C3=O)=C3C(O)=C1 |
| ***Genistein*** | O=C1C(C2=CC=C(C=C2)O)=COC3=CC(O)=CC(O)=C13 |
| ***Hesperidin*** | O=C1C[C@@](C2=CC=C(C(O)=C2)OC)(OC3=CC(O[C@@]([C@H](O)[C@@H](O)[C@H](O)[C@]4(CO[C@@]([C@H](O)[C@H](O)[C@@H](O)[C@@H]5C)(O5)[H])[H])(O4)[H])=CC(O)=C13)[H] |
| ***Hyperoside*** | OC1=C(O)C=CC(C2=C(O[C@H]3[C@H](O)[C@@H](O)[C@@H](O)[C@@H](CO)O3)C(C4=C(O)C=C(O)C=C4O2)=O)=C1 |
| ***Isorhamnetin*** | O=C(C(O)=C(C1=CC=C(O)C(OC)=C1)O2)C3=C2C=C(O)C=C3O |
| ***Kaempferol*** | OC1=CC=C(C2=C(C(C3=C(C=C(C=C3O2)O)O)=O)O)C=C1 |
| ***Kaempferol-3-O-glucoside*** | OC1=CC(O)=C2C(OC(C3=CC=C(C=C3)O)=C(C2=O)O[C@H]([C@H]([C@@H]4O)O)O[C@H]([C@@H]4O)CO)=C1 |
| ***Kaempferol-3-O-rutinoside*** | OC(C=C1)=CC=C1C(OC2=C3C(O)=CC(O)=C2)=C(O[C@H]4O[C@@H](CO[C@@H]5[C@@H](O)[C@@H](O)[C@H](O)[C@@H](C)O5)[C@H](O)[C@@H](O)[C@@H]4O)C3=O |
| ***Luteolin*** | OC1=CC2=C(C(O)=C1)C(C=C(C3=CC=C(C(O)=C3)O)O2)=O |
| ***Luteolin-7-O-glucoside*** | O=C1C=C(OC2=C1C(O)=CC(O[C@](O[C@@H]([C@H]([C@@H]3O)O)CO)([C@@H]3O)[H])=C2)C4=CC(O)=C(O)C=C4 |
| ***Morin*** | OC1=CC=C(C(OC2=C3C(O)=CC(O)=C2)=C(O)C3=O)C(O)=C1 |
| ***Myricetin*** | OC1=C(C(C=C2O)=CC(O)=C2O)OC3=CC(O)=CC(O)=C3C1=O |
| ***Naringenin*** | OC1=CC(O)=CC2=C1C(C[C@@H](C3=CC=C(O)C=C3)O2)=O |
| ***Quercetin*** | OC1=C(C(C=C2)=CC(O)=C2O)OC3=CC(O)=CC(O)=C3C1=O |
| ***Quercitrin*** | OC1=CC(O)=C2C(OC(C3=CC=C(O)C(O)=C3)=C(O[C@H]4[C@H](O)[C@H](O)[C@@H](O)[C@H](C)O4)C2=O)=C1 |
| ***Rhamnetin*** | O=C(C(O)=C(C1=CC(O)=C(O)C=C1)O2)C3=C2C=C(OC)C=C3O |
| ***Rutin*** | C[C@H]([C@H]1O)O[C@@H]([C@H]([C@H]1O)O)OC[C@@H]([C@@H]2O)O[C@@H]([C@H]([C@@H]2O)O)OC3=C(C4=CC=C(C(O)=C4)O)OC5=CC(O)=CC(O)=C5C3=O |
| ***Taxifolin*** | O[C@H]1[C@H](C(C=C2)=CC(O)=C2O)OC3=CC(O)=CC(O)=C3C1=O |
| ***Vitexin*** | O[C@H]1[C@@H]([C@H]([C@H](CO)O[C@@H]1C2=C(O)C=C(O)C3=C2OC(C4=CC=C(C=C4)O)=CC3=O)O)O |

**
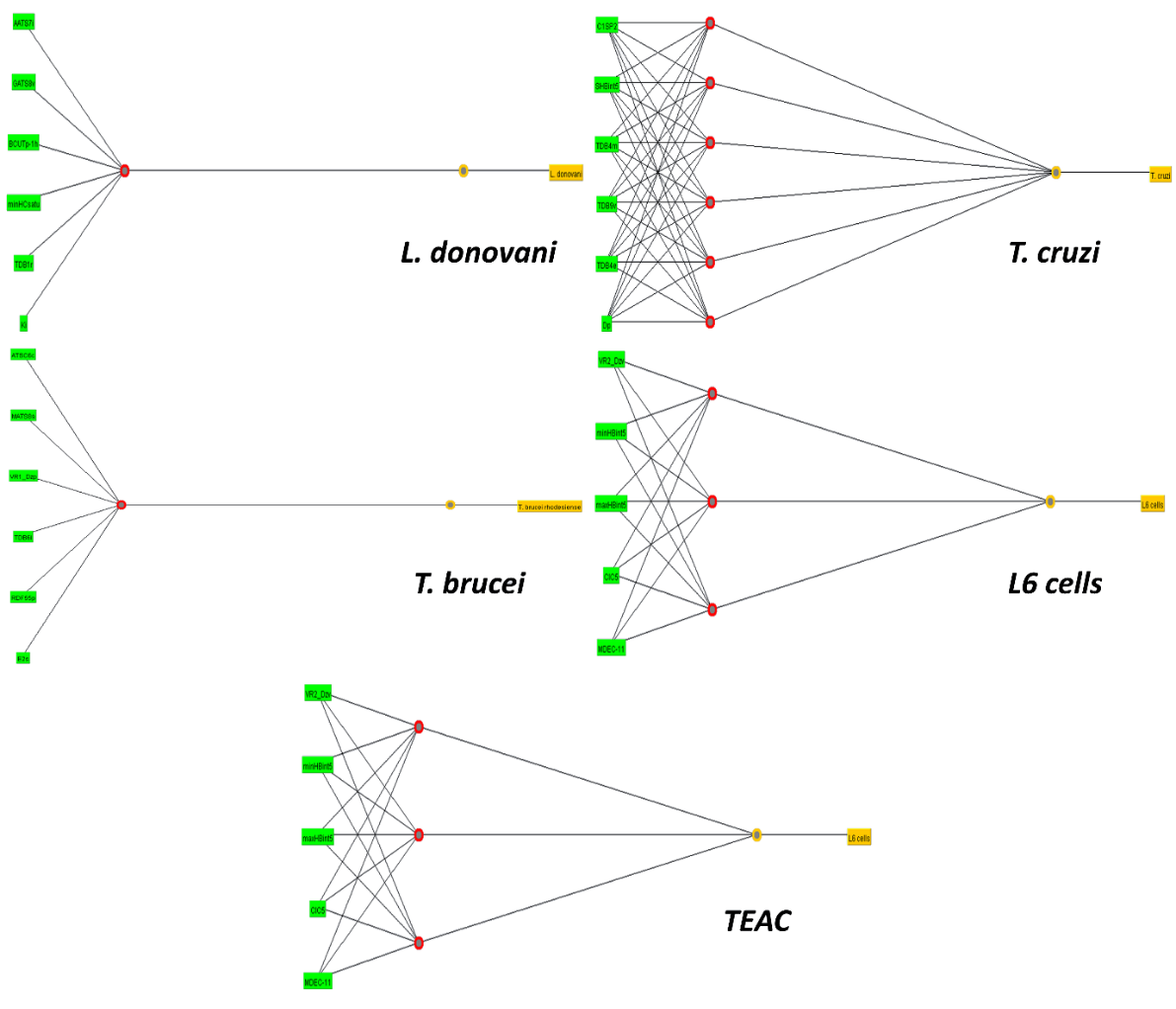
**

**Supplementary Figure S1.** Artificial Neural Networks (ANNs) built on Weka 3.8.0 using the classifier Multilayer Perceptron. They have applicability domain for prediction of IC_50_ of flavonoids in *L. donovani* (R^2^= 0.75, Q^2^= 0.62, P^2^= 0.67), *T. cruzi* (R^2^= 0.94, Q^2^= 0.67, P^2^= 0.92), *T. brucei* (R^2^= 0.93, Q^2^= 0.73, P^2^= 0.66) and TEAC (R^2^= 0.89, Q^2^= 0.72, P^2^ = 0.66). All of them show R^2^, Q^2^ and P^2^ lower than 0.41 for scramble test. Except ANN built to predict toxicity against L6 cells that not show predictive ability even robustness (R^2^= 0.63, Q^2^= 0.33, P^2^ = 0.48).
