# Supplementary figures and images for: The Correlation between Chemical Structures and Antioxidant, Prooxidant, and Antitrypanosomatid Properties of Flavonoids
Source: Oxid Med Cell Longev. 2017 Jul 2;2017:3789856. doi: 10.1155/2017/3789856 (PMC5511661; doi:10.1155/2017/3789856)

## Slide 1
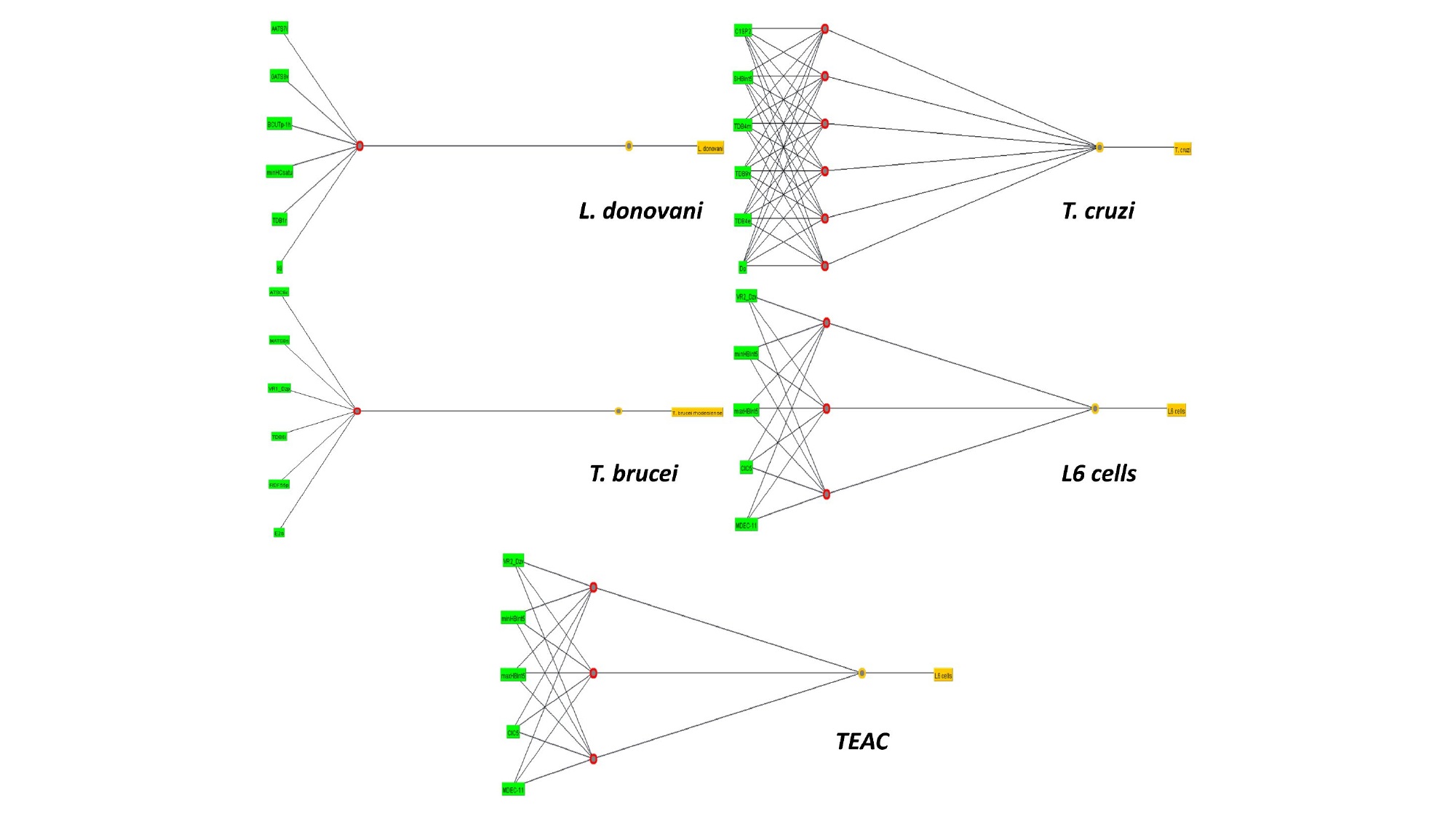

Supplement: Supplementary file 2 [file 3789856.f2.pptx]
